# Supplementary material for: Impact of DNA methylation on 3D genome structure
Source: Nat Commun. 2021 May 28;12:3243. doi: 10.1038/s41467-021-23142-8 (PMC8163762; doi:10.1038/s41467-021-23142-8)
Supplement: Supplementary file 1 — Supplementary Information [file 41467_2021_23142_MOESM1_ESM.pdf]

SUPPLEMENTARY INFORMATION

Impact of DNA methylation on 3D genome structure

Buitrago et al.

- Content

  - Supplementary Figures 1 to 19
  - Supplementary Tables 1 to 5
  - Supplementary Methods

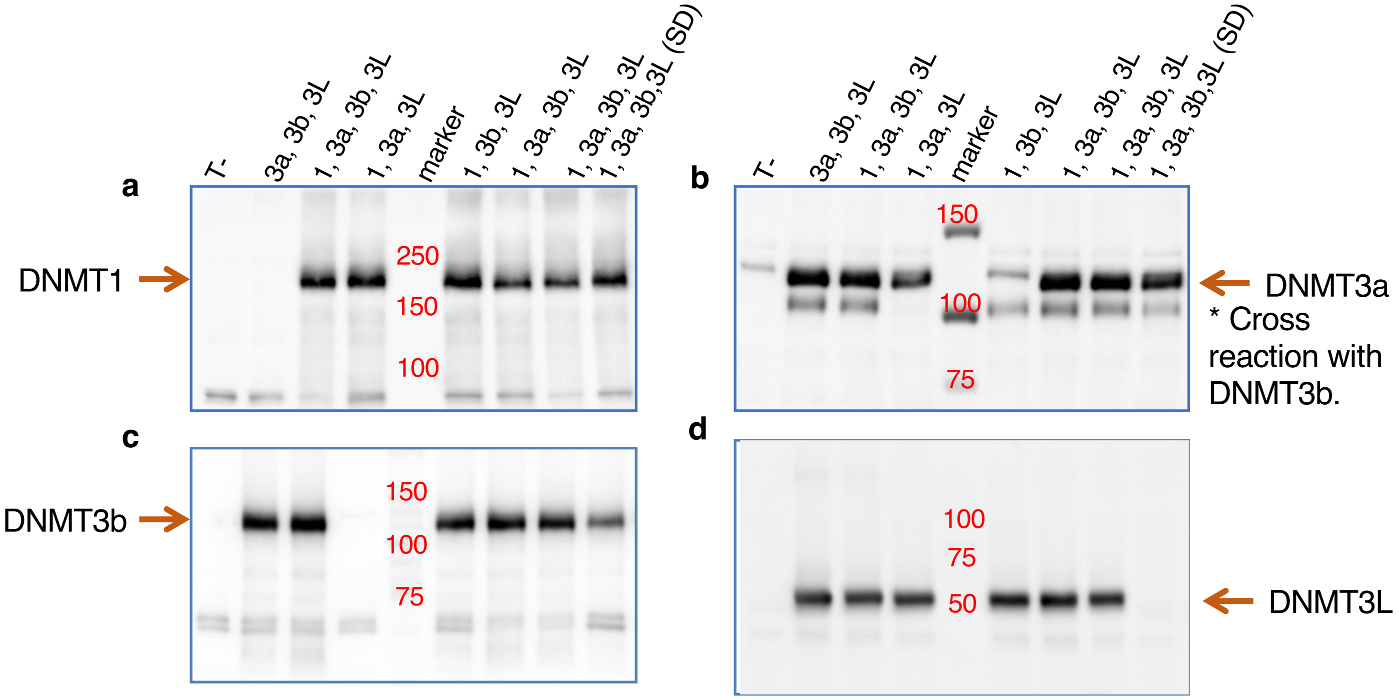

Supplementary Figure 1. Expression and stability of the 4 DNMTs. (**a-d**) The lysate from 7 transformants expressing different combinations of the 4 DNMTS, and one control (T-) were loaded in (**a**) 6% (B,C) 8% and **(d)** 10% acrylamide gel, transferred onto PVDF membrane and revealed with **(a)** anti DNMT1 antibody, (**b**) anti-DNMT3a (**c**) anti DNMT3b and (**d**) anti-Flag antibody. MW (in Da) of the marker bands (All Blue, BIO-RAD) are indicated in red on the pictures. Uncropped gels are provided as Source Data file.

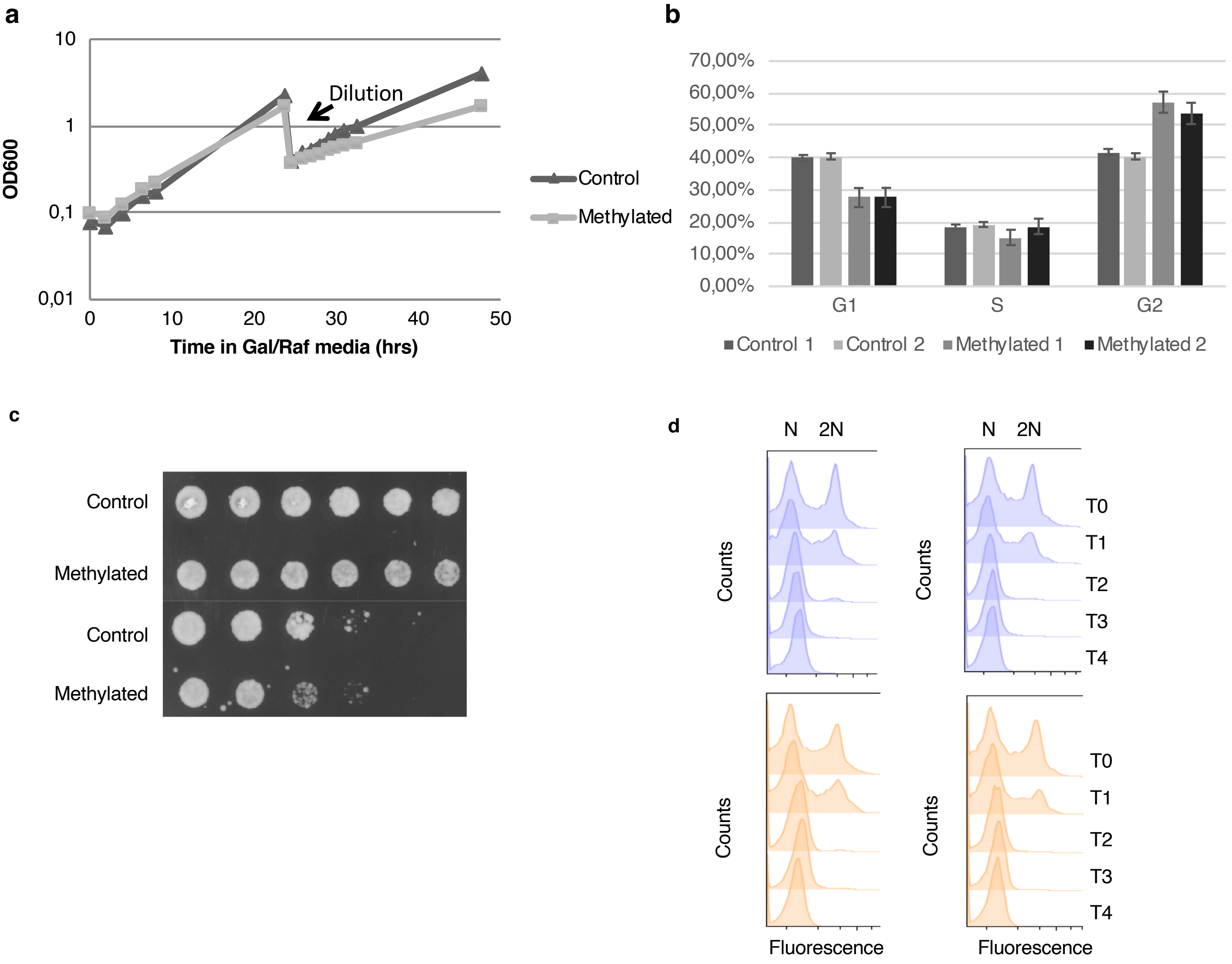

Supplementary Figure 2. (**a**) Representative growth curves obtained from one culture of cells transformed with empty vectors (Control) or cells expressing DNMTs (Methylated) after transfer in inducible media (Galactose + Raffinose) (**b**) Flow cytometry analysis of two independent yeast cultures transformed with empty vectors (Control) and two independent yeast cultures expressing DNMTs (Methylated) in exponential phase. The gating strategy is presented in Supplementary Fig. 19a-h. A Dirichlet-multinomial model was fitted separately to the control replicates and the methylation replicates, with the parameters being estimated by maximum likelihood. The expected standard errors (se) for each estimate of the cell type percentage were calculated from the Dirichlet-multinomial model using the multiple likelihood estimates, and the 95% limits were then constructed as +/- 1.96 * se. (**c**) Viability test of a yeast culture transformed with empty vectors (Control) and one expressing the 4 DNMTs (Methylated). Test performed doing 2x serial dilutions (Top) or 10x serial dilutions (Bottom). (**d**) Flow cytometry analysis of two independent yeast cultures transformed with empty vectors (Top 2 plots in violet) and two independent yeast cultures expressing DNMTs (Bottom 2 plots in orange). Time-course after induction of DNMTs expression in exponential phase at OD_{600}=0.5 (control) and OD_{600}=1 (methylated) (T0) and 12 hours (T1, OD_{600}=1.7 to 2.2), 24 hours (T2, OD_{600}=4 to 5), 34 hours (T3, OD_{600}=5.8 to 7) and 48 hours later (T4, OD_{600}=6 to 7). The gating strategy is similar to the one used for Supplementary Fig2b and presented in Supplementary Fig. 3. Cell cycle distribution was determined using FlowJo software.

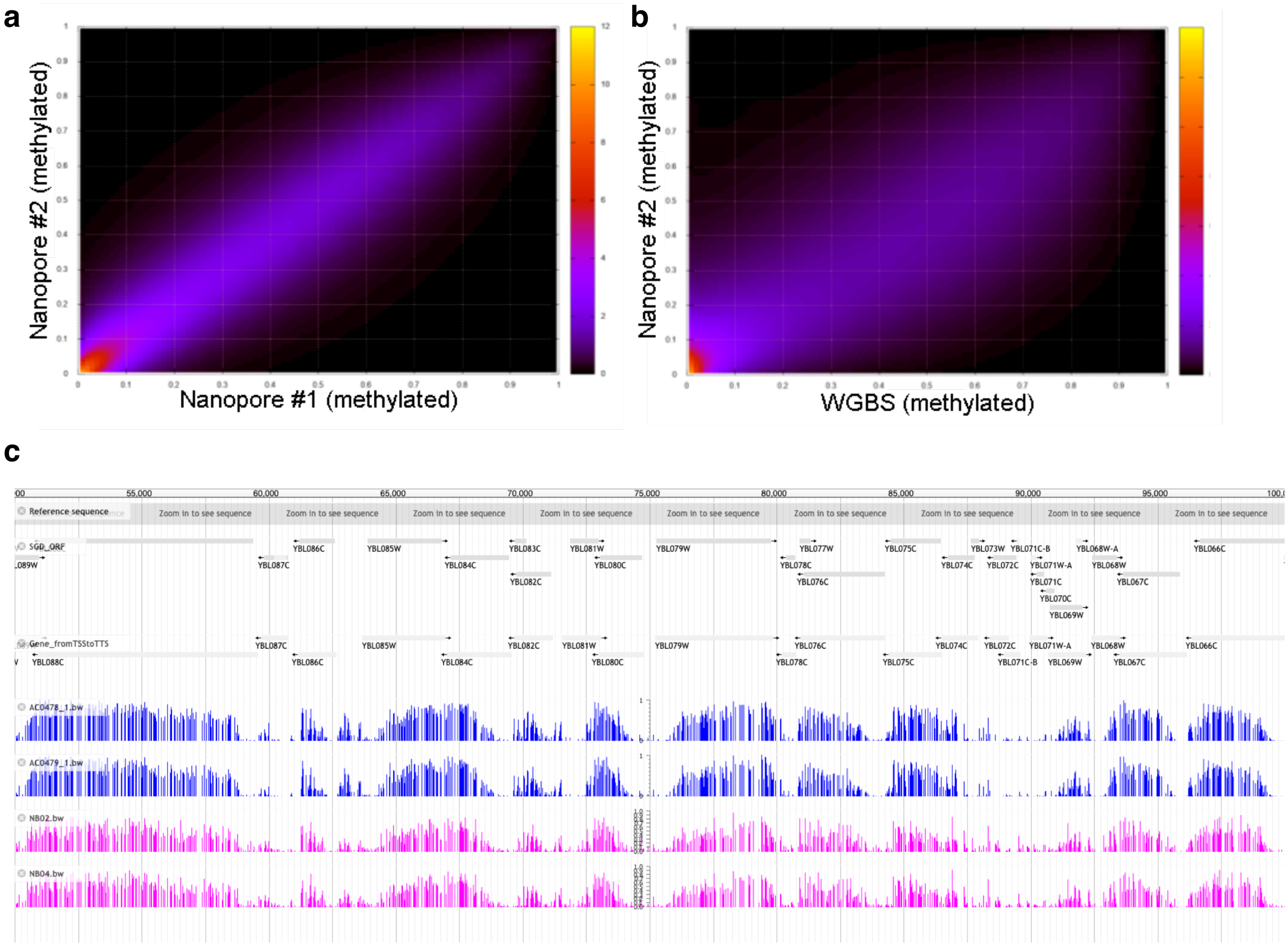

Supplementary Figure 3. Heatmap showing the pairwise CpG methylation correlation in (**a**) two nanopore replicates and (**b**) in one nanopore vs one WGBS replicate. (**c**) Methylation pattern for a 50kb region of chromosome II (50,000-100,000) in WGBS (top 2 tracks in blue) and nanopore (bottom tracks in magenta) samples for 2 replicas of each condition.

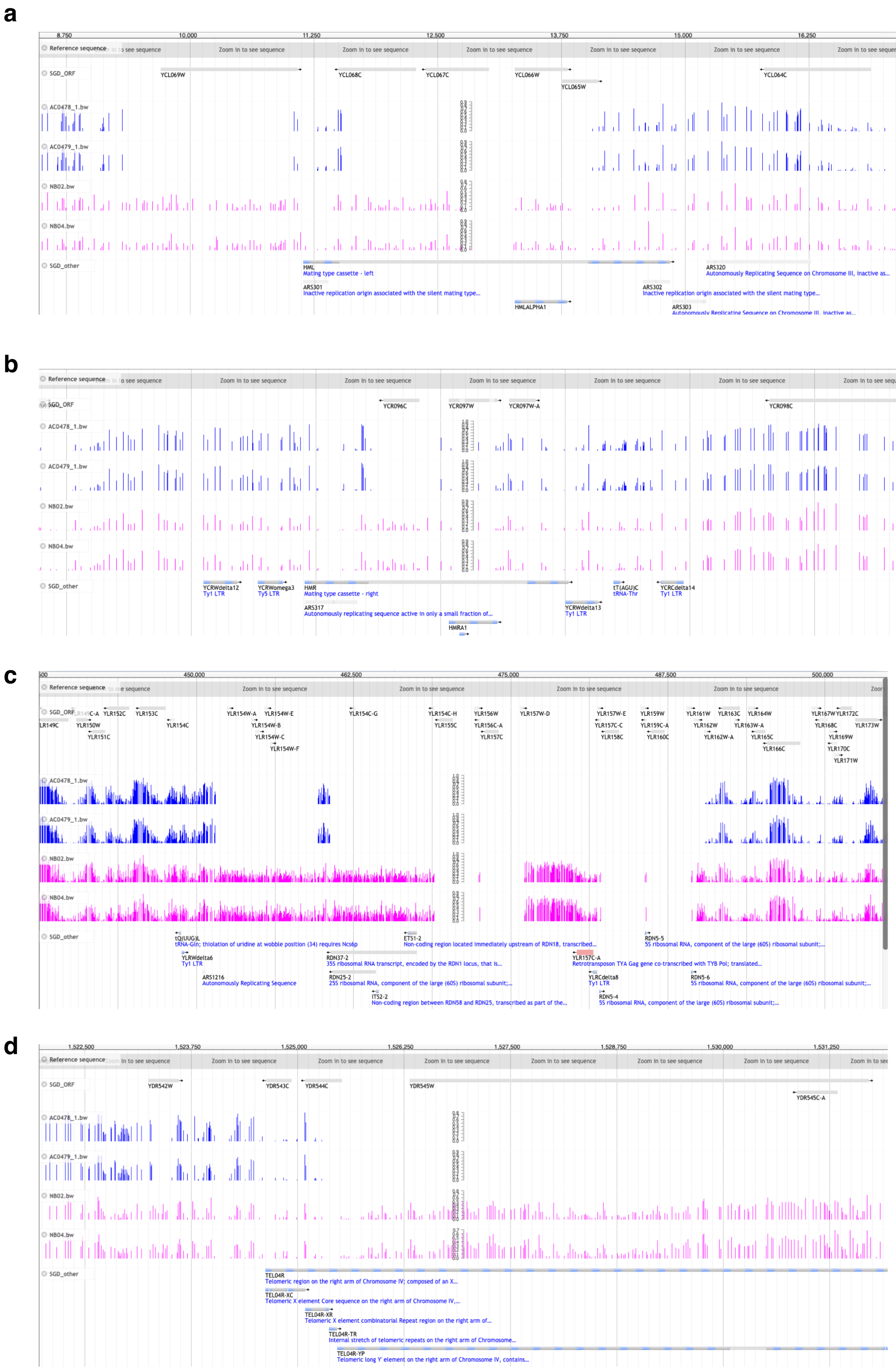

Supplementary Figure 4. Methylation pattern from WGBS (top tracks in blue) and nanopore (bottom tracks in magenta) samples for 2 biological replicates at (**a**) the HML locus, (**b**) the HMR locus, (**c**) the rDNA locus and (**d**) a telomeric region in chromosome IV.

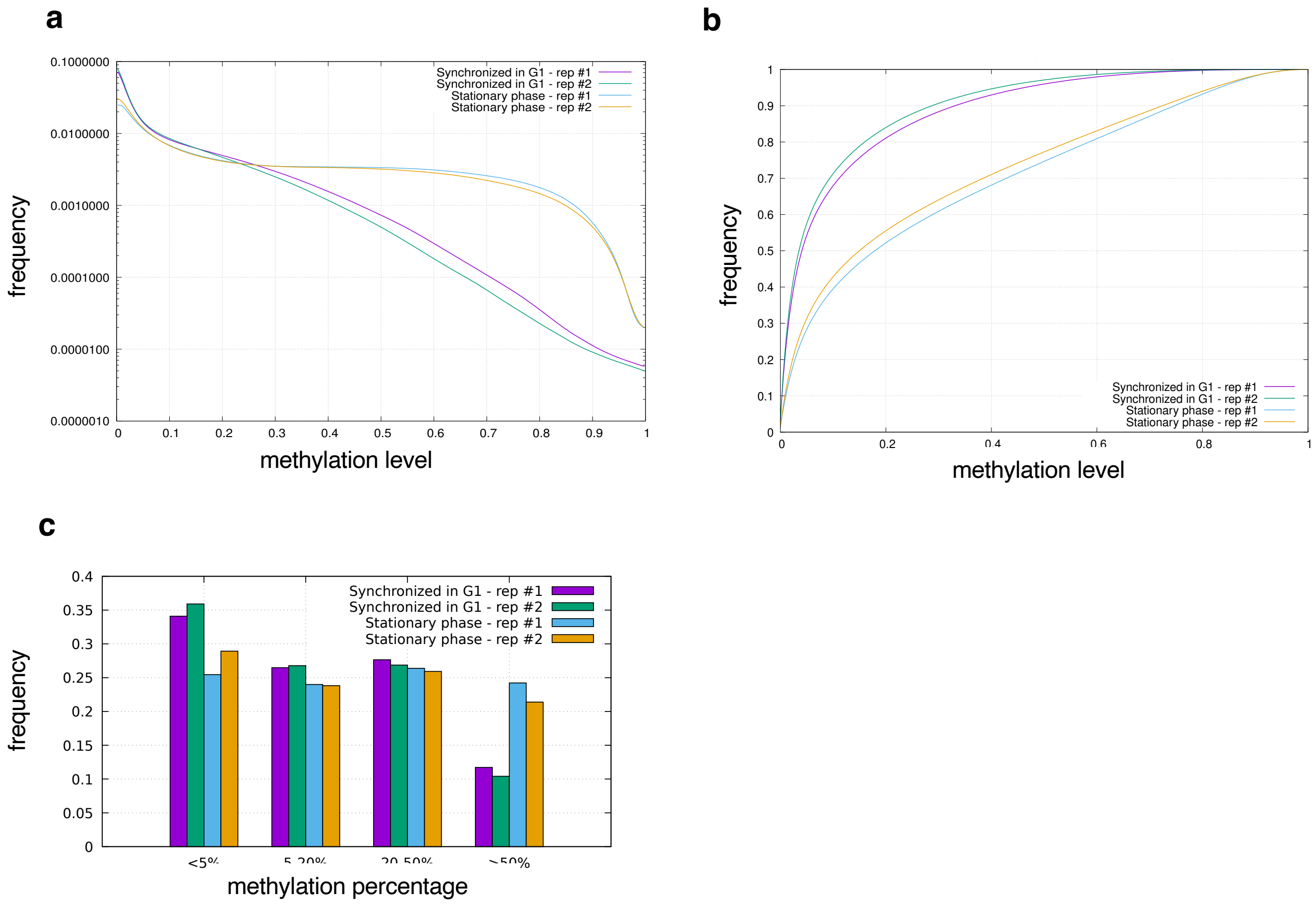

Supplementary Figure 5. Global methylation levels across all CpG sites from WGBS in two exponential cultures synchronized in G1 and two stationary cultures (**a**) Distribution of methylation values. (**b**) Cumulative distribution of methylation values. (**c**) Histogram showing the % of all CpG sites with methylation in the ranges <5%, 5-20%, 20-50% and >50%.

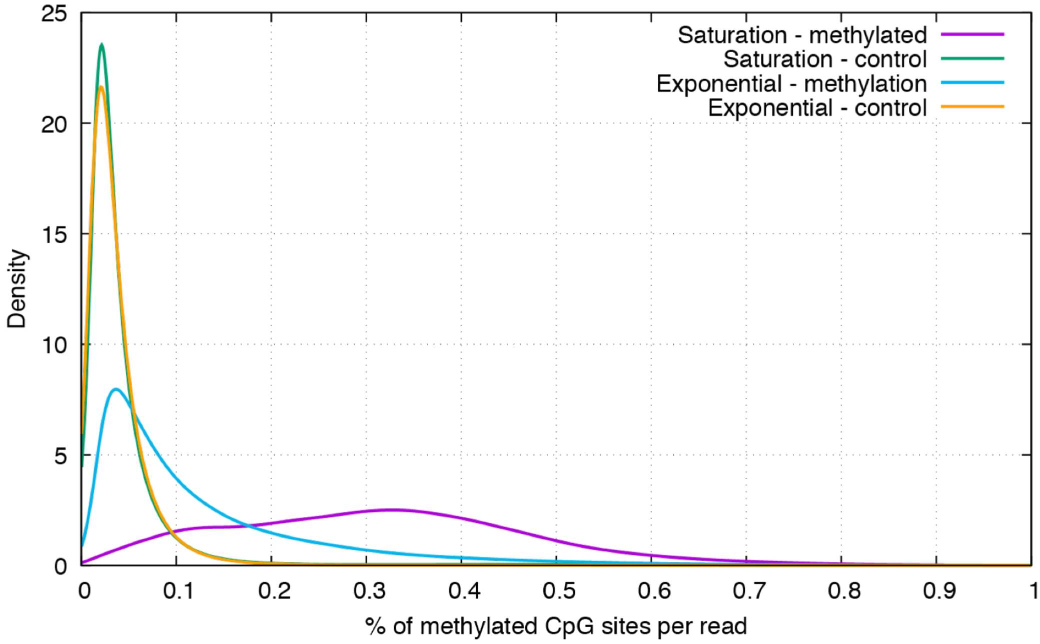

Supplementary Figure 6. Density estimates of the % of CpG sites methylated per read estimated from Nanopore sequence data for four datasets: methylated cells in stationary phase, control cells in stationary phase, methylated cells in exponential phase and control cells in exponential phase.

a

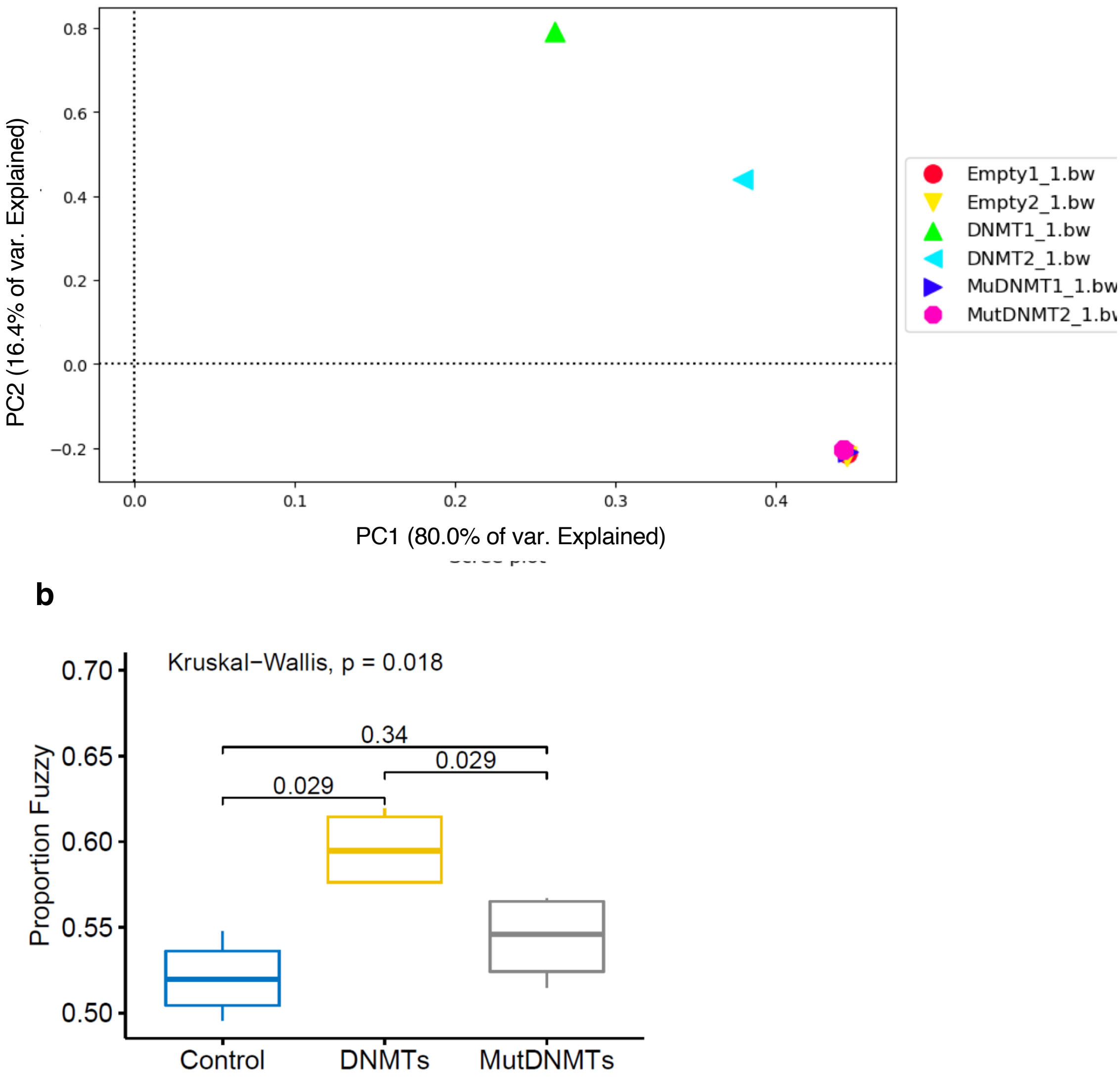

Supplementary Figure 7. Comparison of nucleosome profiles in the control, methylated, and inactive DNMTs strains. (**a**) Principal component analysis comparing nucleosome coverage profiles in the three conditions. (**b**) Boxplot of the proportion of fuzzy nucleosomes per condition. Wilcoxon two-sided test comparing the pairwise difference in the proportion of fuzzy nucleosomes is shown on top of the boxplots. Boxplots lower and upper hinges correspond to the first and third quartiles, respectively, and the middle line represents the median. The upper whisker extends from the hinge to the largest value no further than 1.5 × IQR from the hinge (where IQR is the inter-quartile range) and the lower whisker extends from the hinge to the smallest value at most 1.5 × IQR of the hinge.

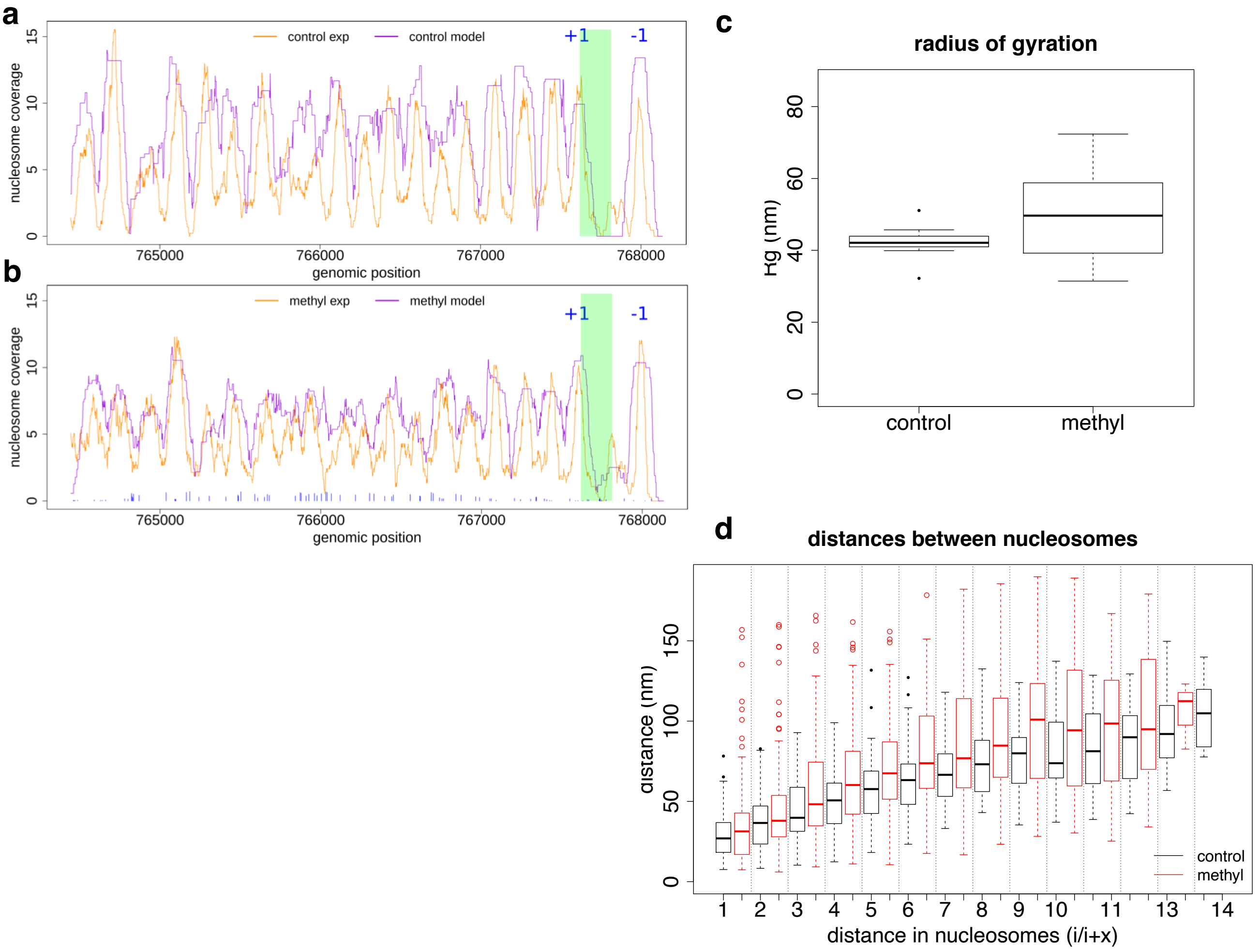

Supplementary Figure 8. Modeled nucleosome fibers from MNase-seq data. (**a**) Nucleosome position coverage for DUG2 gene (chromosome II) in control cells (experimental and modeled). The TSS is highlighted in green and nucleosomes -1 and +1 are indicated. (**b**) Nucleosome position coverage for DUG2 gene (chromosome II) in methylated cells (experimental and modeled). The TSS is highlighted in green and nucleosomes -1 and +1 are indicated. The blue sticks in the bottom correspond to methylated regions. Ensemble distribution of (**c**) radius of gyration and (**d**) 3D distances between nucleosomes that are *x* nucleosome units apart for modeled nucleosome fibers. *n* = 1,000 structures. All values are plotted including outliers as dots/circles. The boxes depict the first, second and third quartiles, while whiskers extend no more than 1.5 times the interquartile range from the box limit.

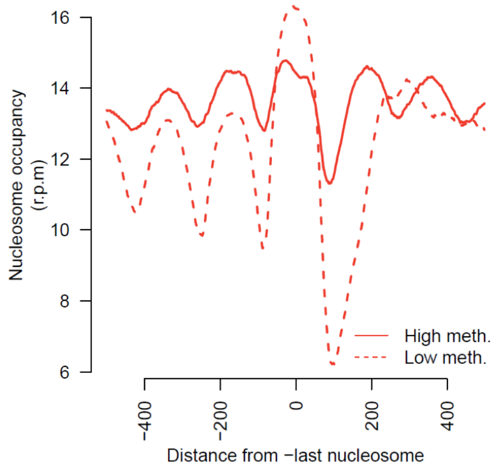

Supplementary Figure 9 : Nucleosome coverage around -last nucleosome for genes with highest (top 10%) or lowest (bottom 10%) methylation level around the TTS.

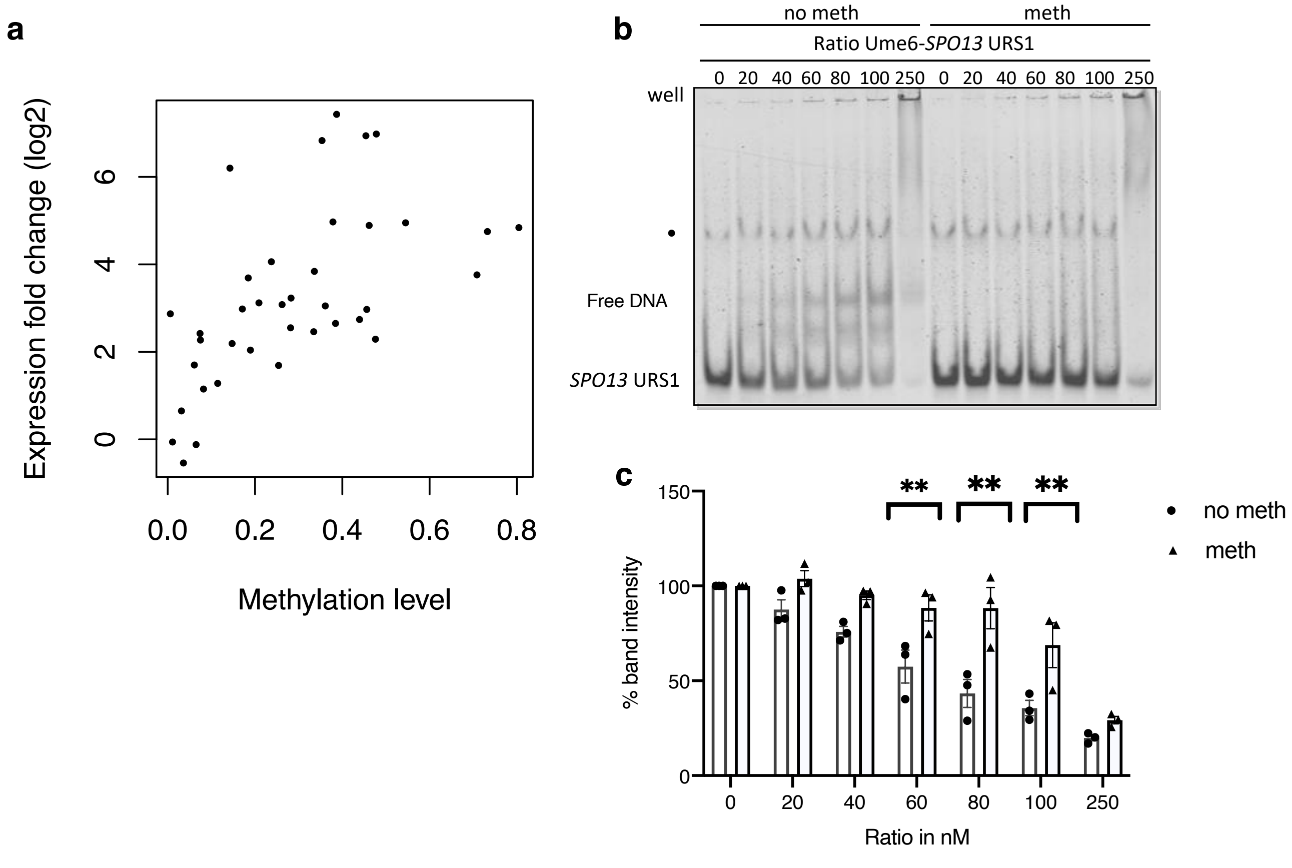

Supplementary Figure 10. (**a**) Expression changes between the control and the methylated samples according to the methylation level for the early meiotic genes listed in Supplementary table 4. (**b**) Gel retardation of URS1 SPO13 sequence with (met) or without (no meth) methylated cytosines upon binding of Ume6. Gel representative of the results obtained in 3 experiments. Uncropped gels are provided as Source Data file. ● Non-specific band. (**c**) Quantification of SPO13 URS1 band from triplicate experiments. Data shows the percentage of unbound DNA compared to the control without Ume6 protein. Error bars are derived from the SEM of the three replicates. Adjusted P value ** $p \leq 0.01$ (Multiple unpaired t test, Ratio60 : Adj.P=0.006, Ratio80 : Adj. P=0.0001 and Ratio 100 : Adj. P=0.004). Source data are provided as a Source Data file

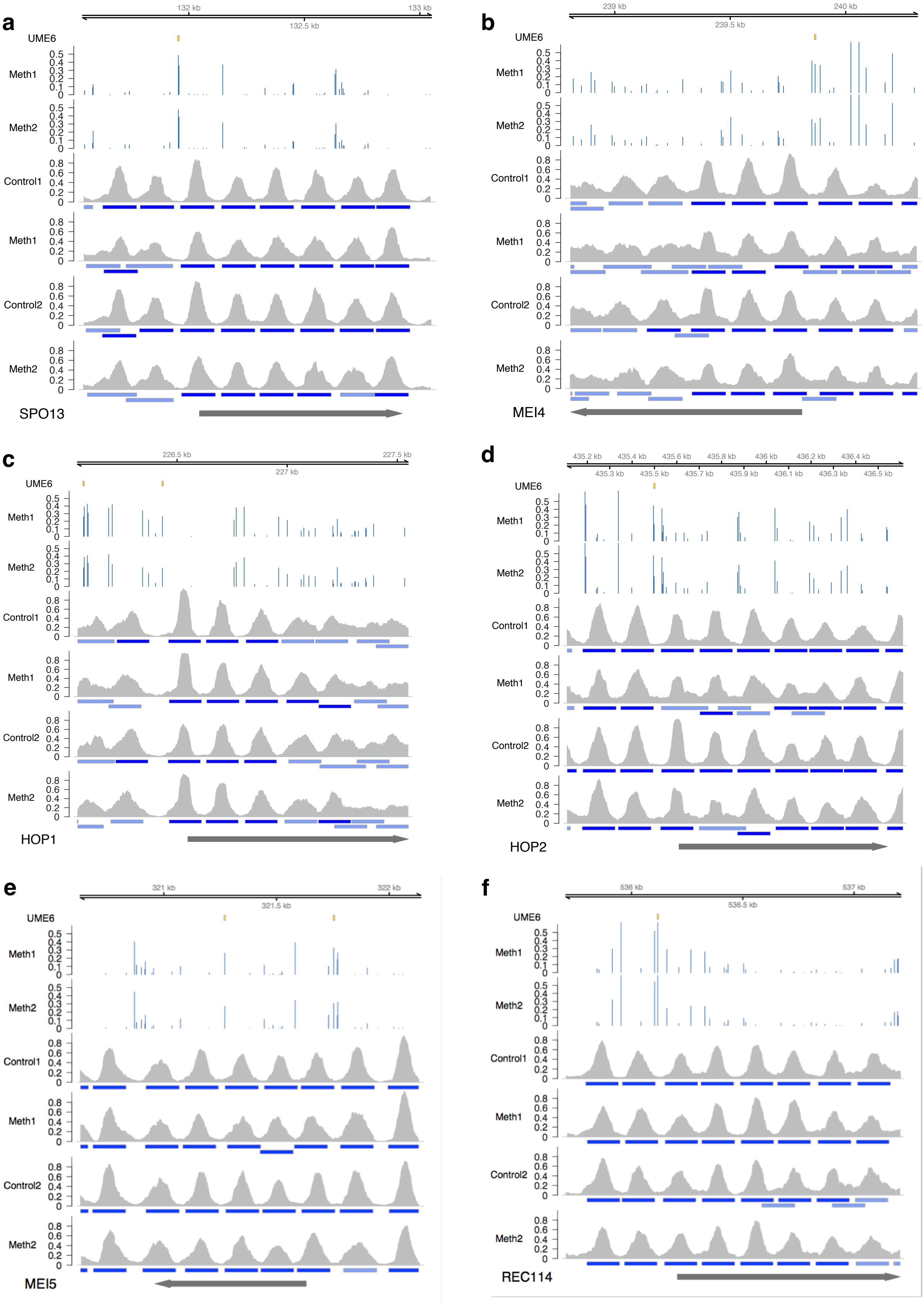

Supplementary Figure 11

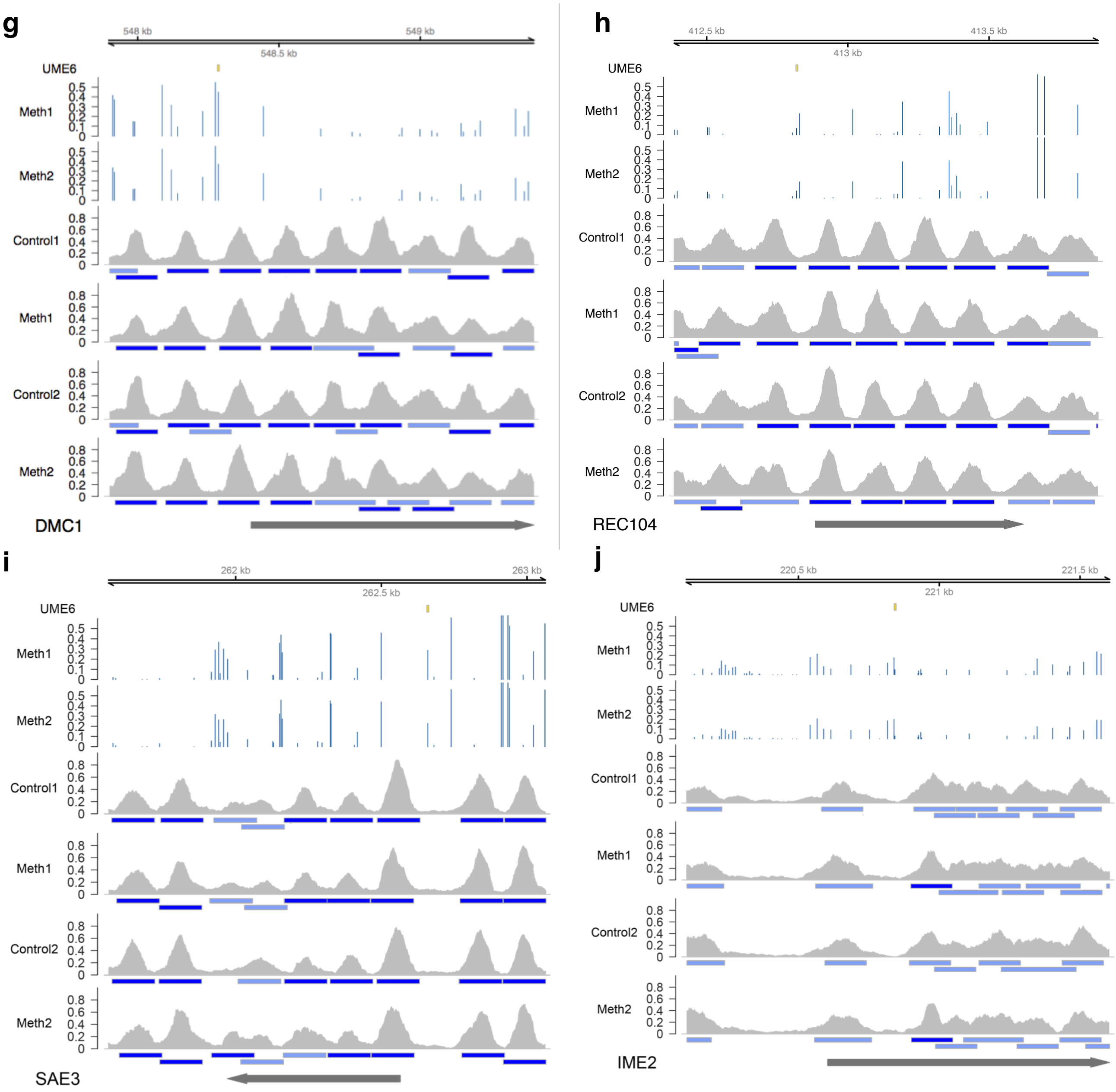

Supplementary Figure 11. Nucleosome position changes upon DNA methylation for 10 upregulated genes. (**a**) SPO13, (**b**) MEI4, (**c**) HOP1, (**d**) HOP2, (**e**) MEI5, (**f**) REC114, (**g**) DMC1, (**h**) REC104, (**i**) SAE3 and (**j**) IME2. The position of UME6 DNA binding sequence is indicated as a yellow box, and the methylation levels at individual CpG as a blue histogram for two replicas (Meth1, Meth2). The blue boxes represent the nucleosomes as called by nucleR for two control and two methylated samples.

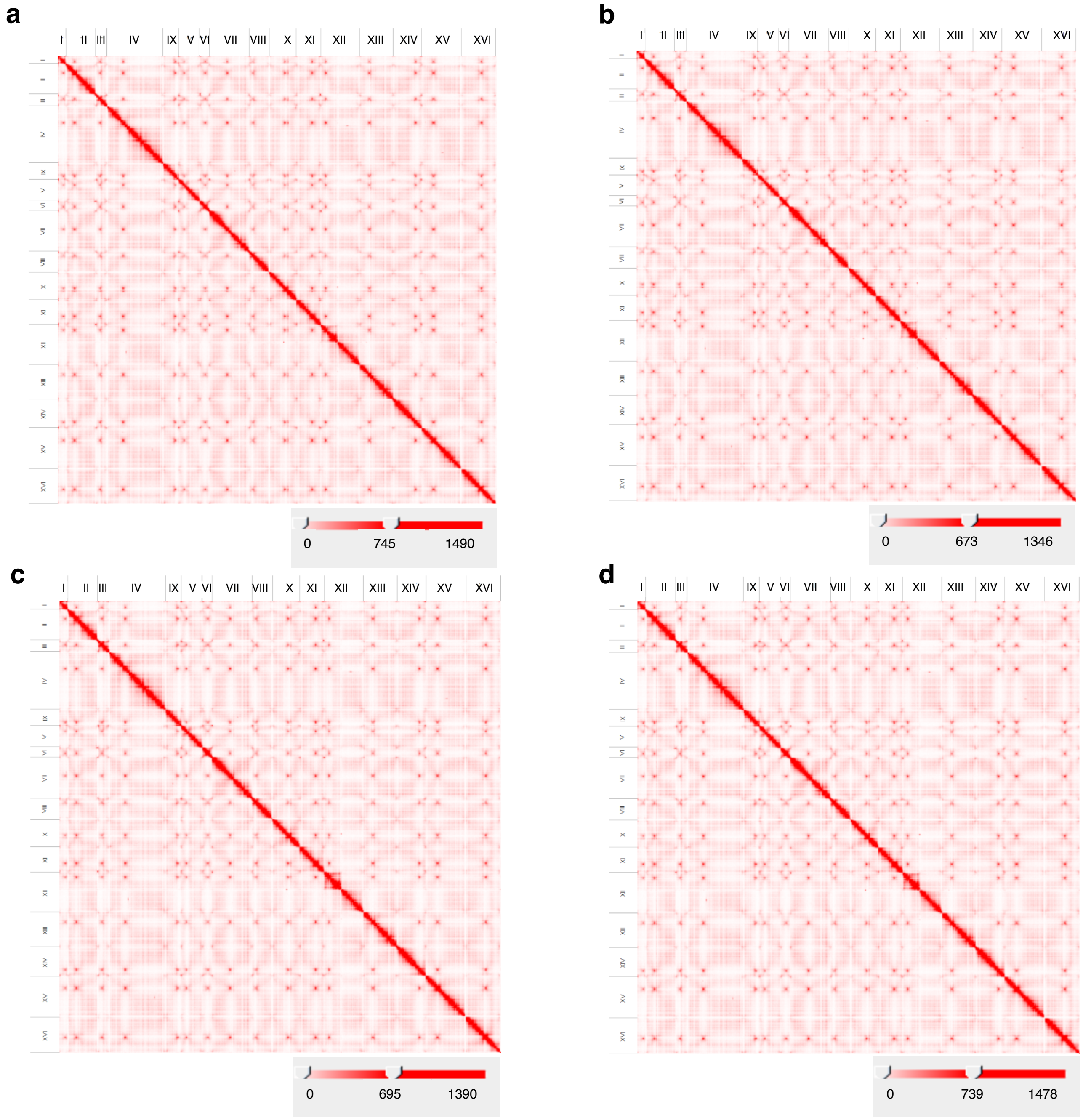

Supplementary Figure S12. Whole genome contact frequency maps in 2 replicates of control (**a,b**) and methylated (**c,d**) samples at 24kb resolution. The intensity of each pixel represents the number of contacts between a pair of loci.

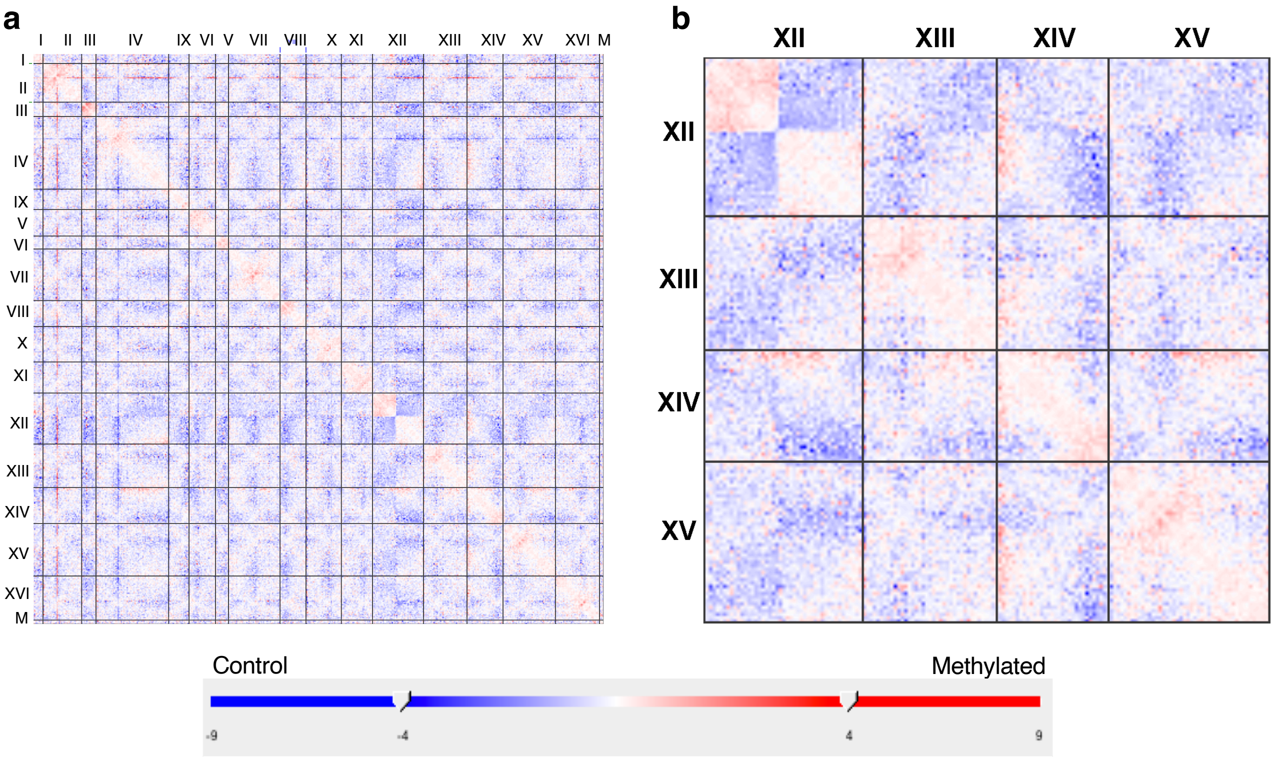

Supplementary Figure 13. Effect of DNA methylation on 3D genome structure in replica 2. Differential contact frequencies in control and methylation induced samples for (**a**) whole genome and (**b**) focus on four chromosomes. Blue indicates interaction with a higher frequency in the non-methylated control sample and red indicates interactions with a higher frequency in the methylated samples.

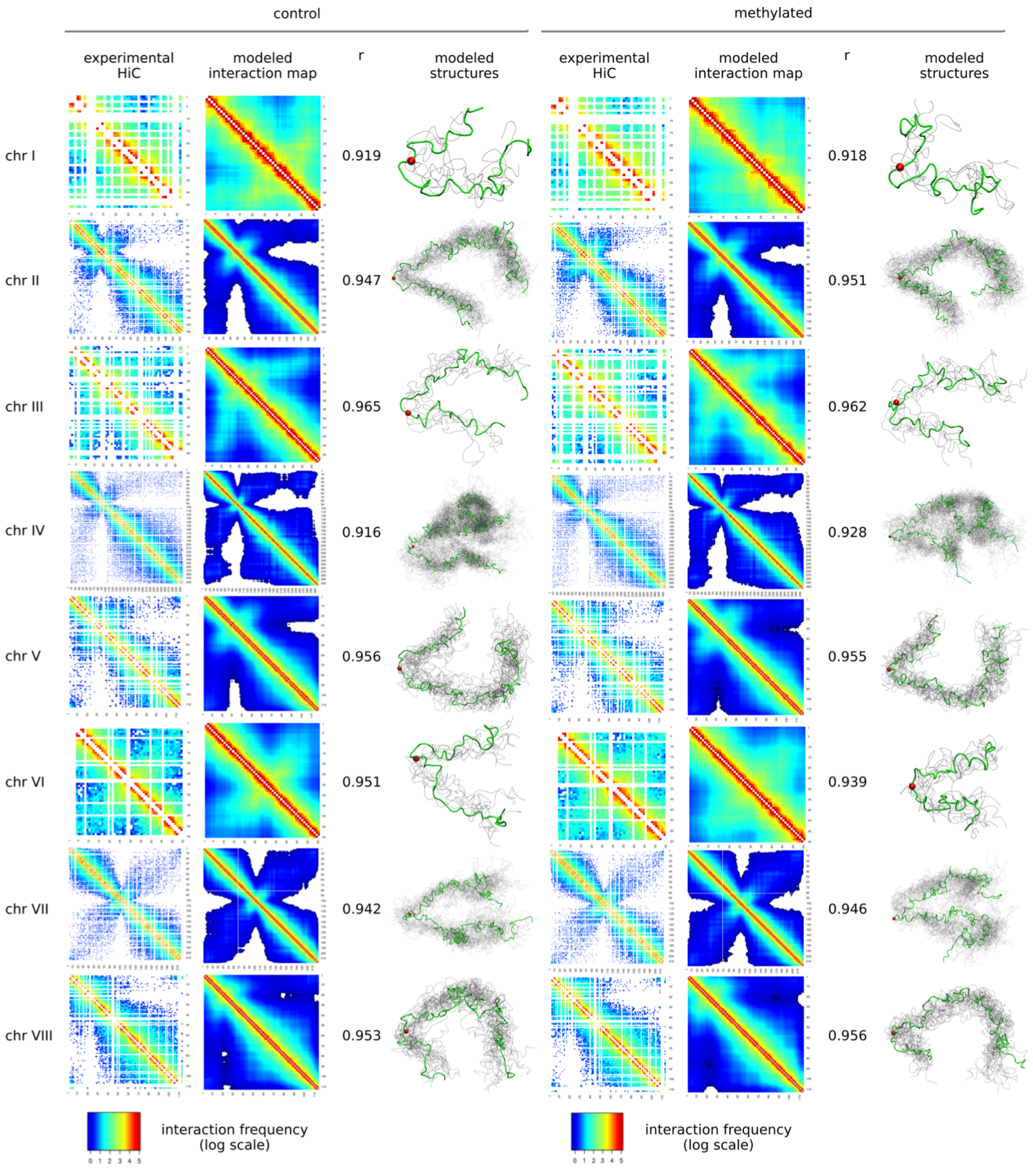

control
methylated
experimental HiC
modeled interaction map
r
modeled structures
chr I
0.919
0.918
chr II
0.947
0.951
chr III
0.965
0.962
chr IV
0.916
0.928
chr V
0.956
0.955
chr VI
0.951
0.939
chr VII
0.942
0.946
chr VIII
0.953
0.956
interaction frequency (log scale)
0 1 2 3 4 5

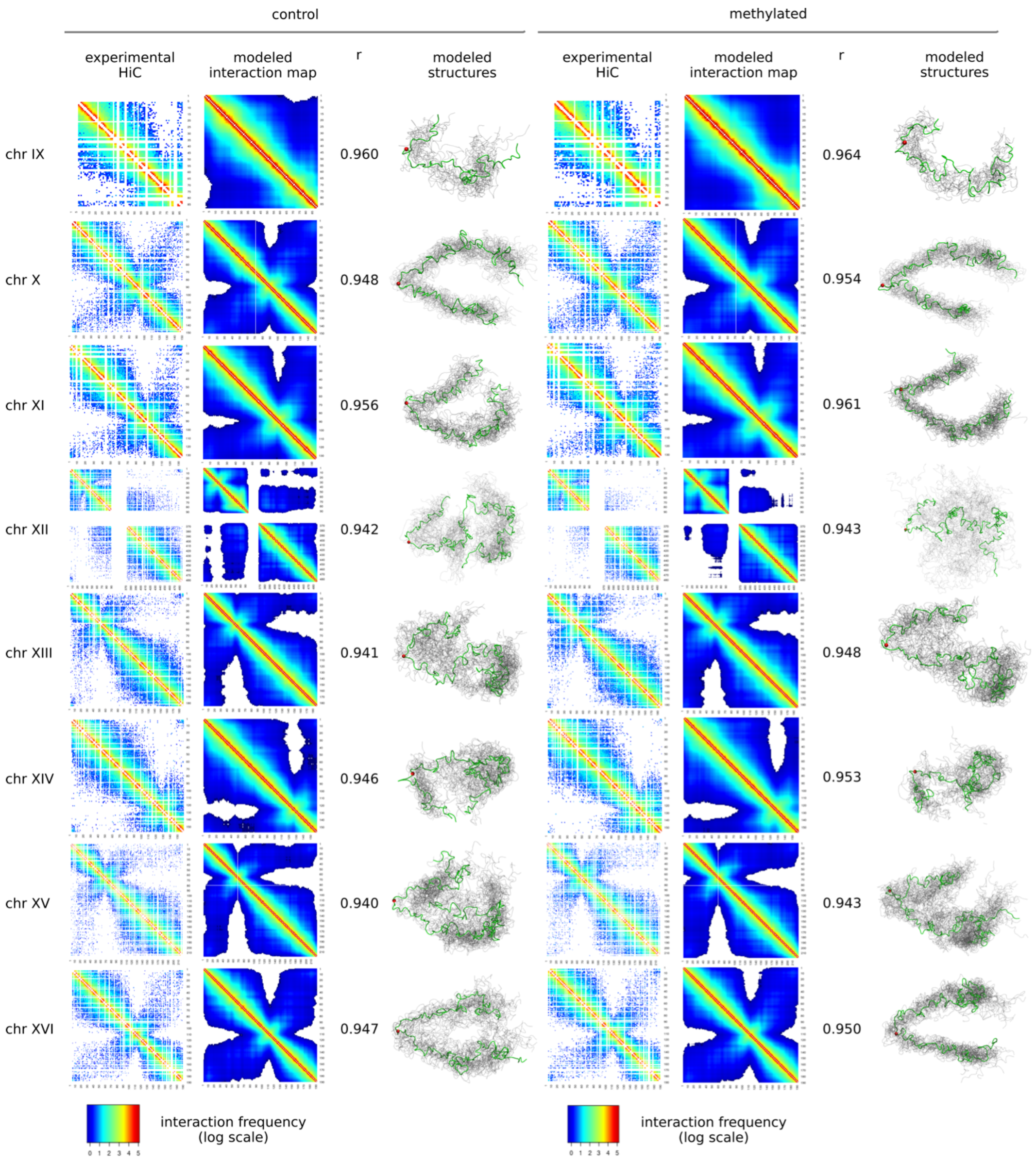

Supplementary Figure 14. Pearson correlation between experimental Hi-C map and mean contact map from ensemble of modeled structures for each chromosome. A representative 3D structure is shown in green with the ensemble shown in translucent gray.

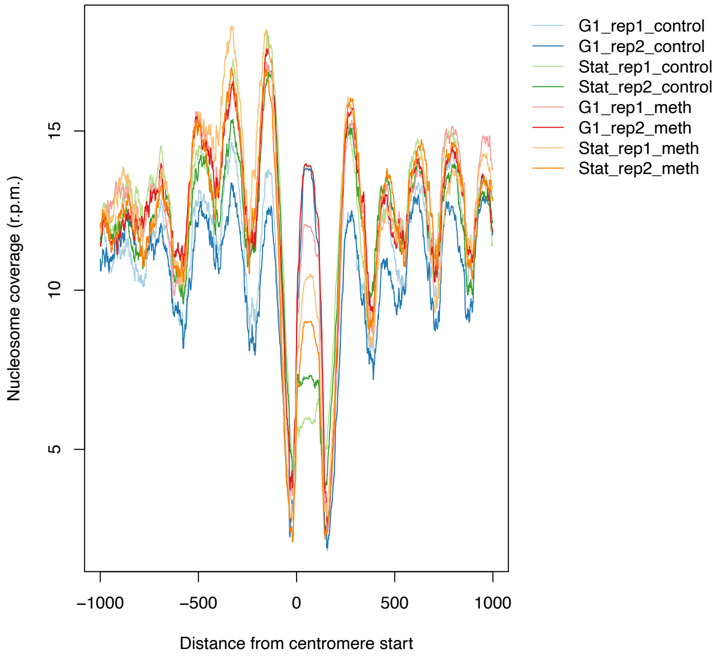

Supplementary Figure 15. Nucleosome coverage at centromeres in G1 and stationary phase.

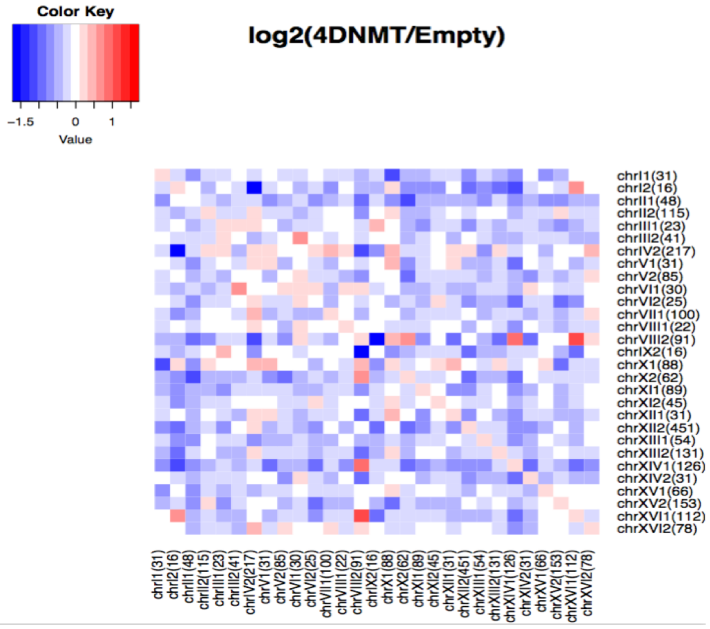

Supplementary Figure 16. Differential contacts between telomeres in the control versus the methylated sample. In general the number of contacts in the control sample is larger (in blue) than in the methylated one (in red).

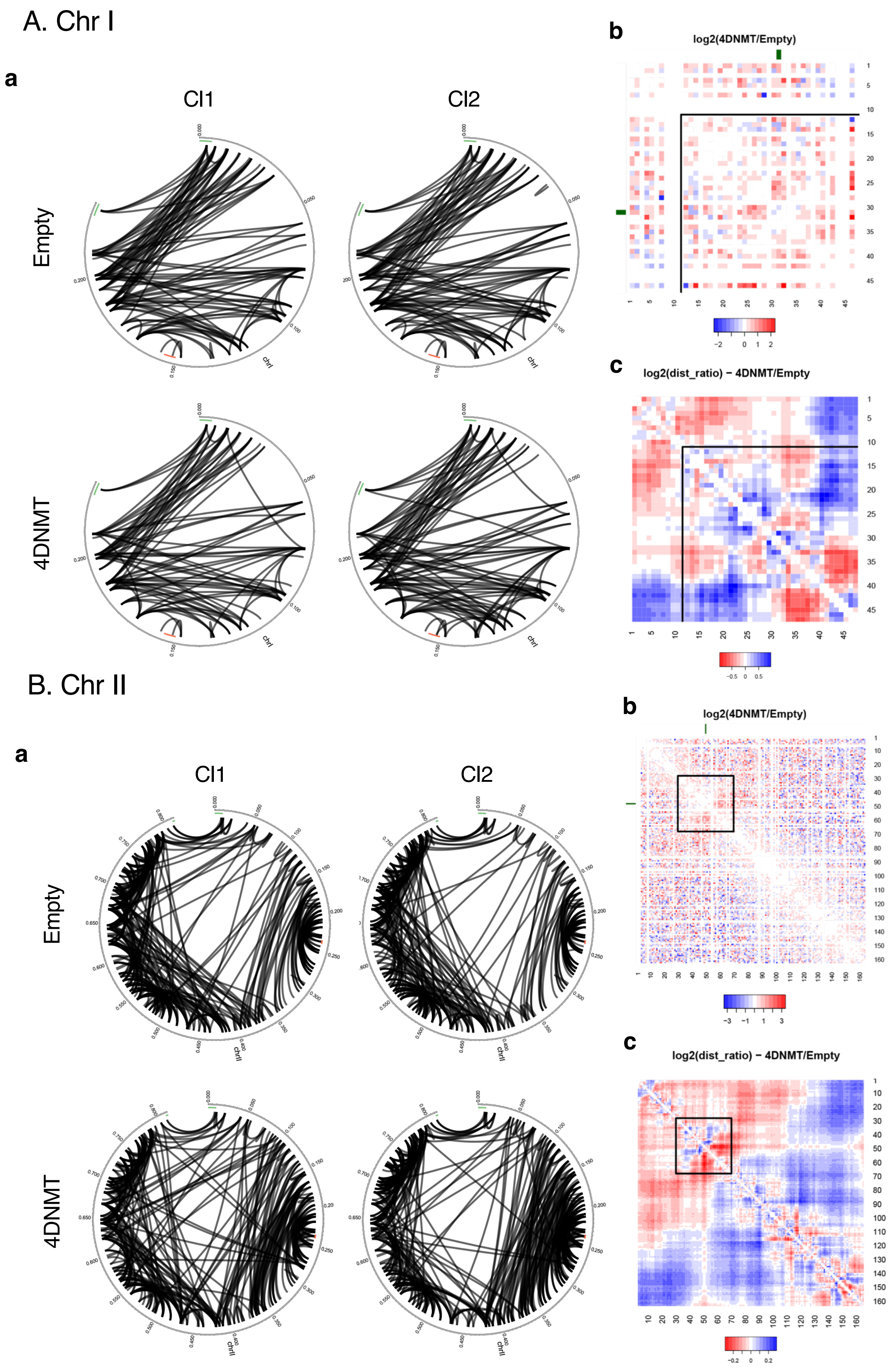

Supplementary Figure 17. Comparison of interactions in the control and the methylated strains

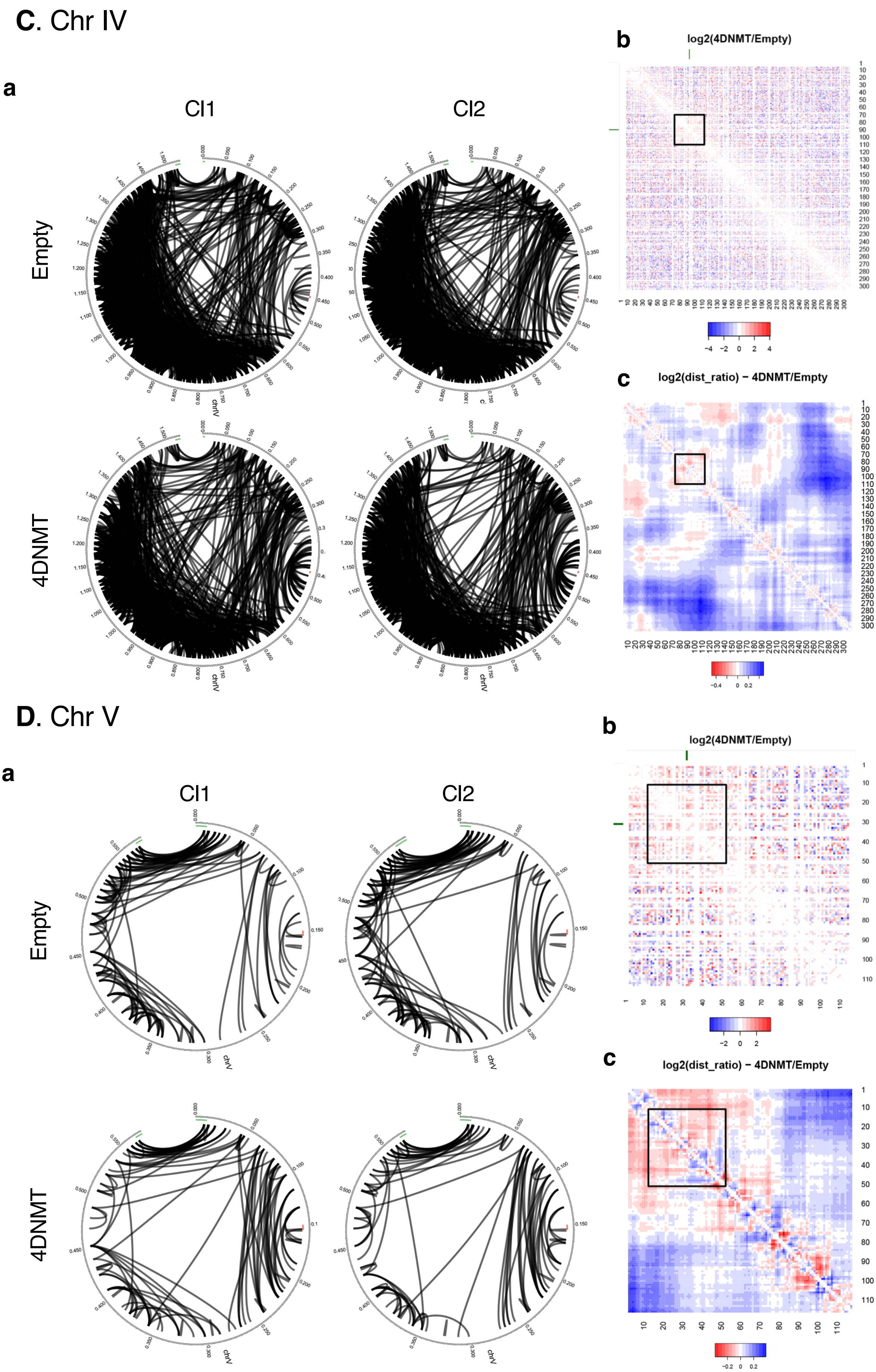

Supplementary Figure 17. Comparison of interactions in the control and the methylated strains

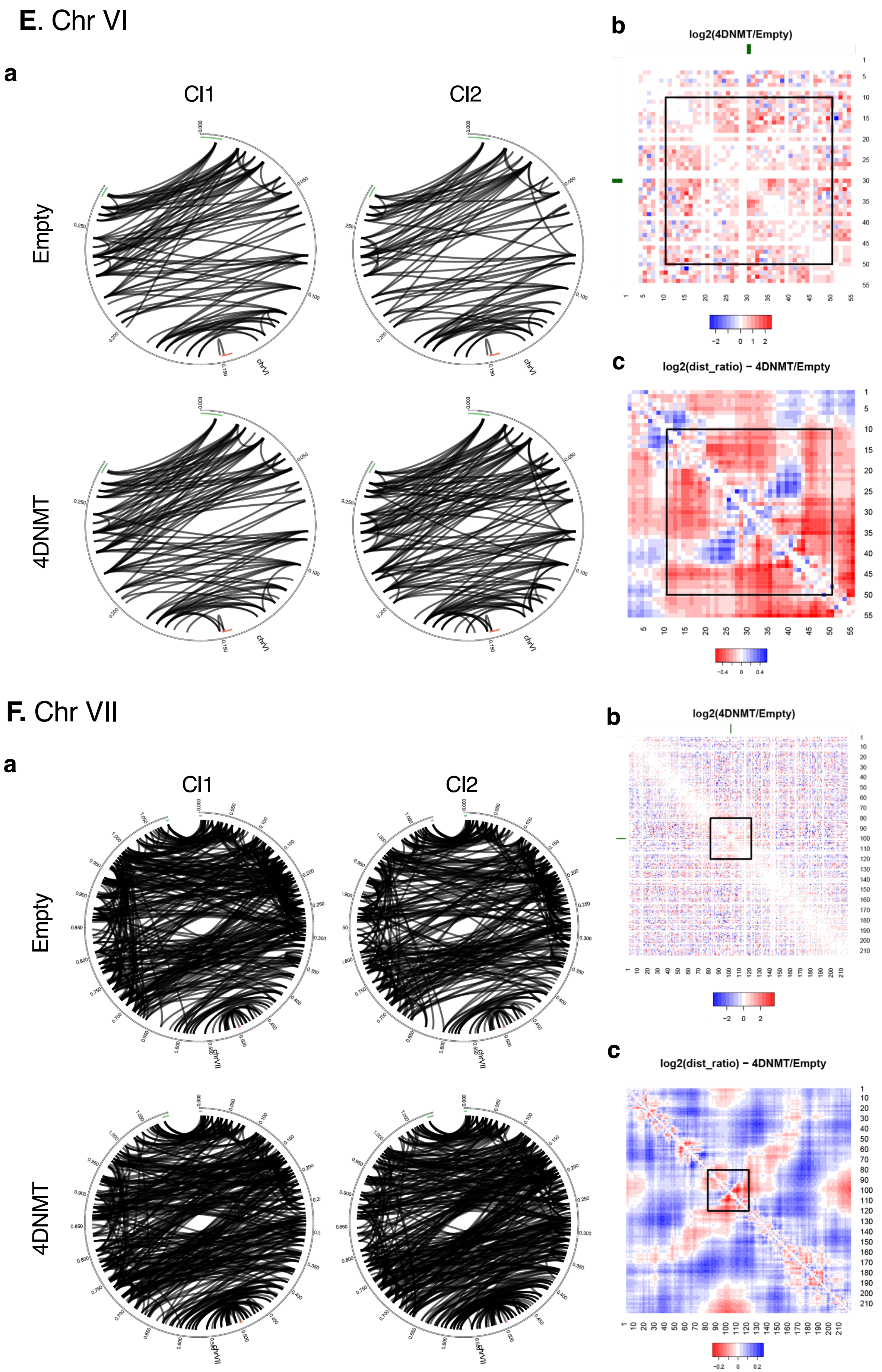

Supplementary Figure 17. Comparison of interactions in the control and the methylated strains

G. Chr VIII

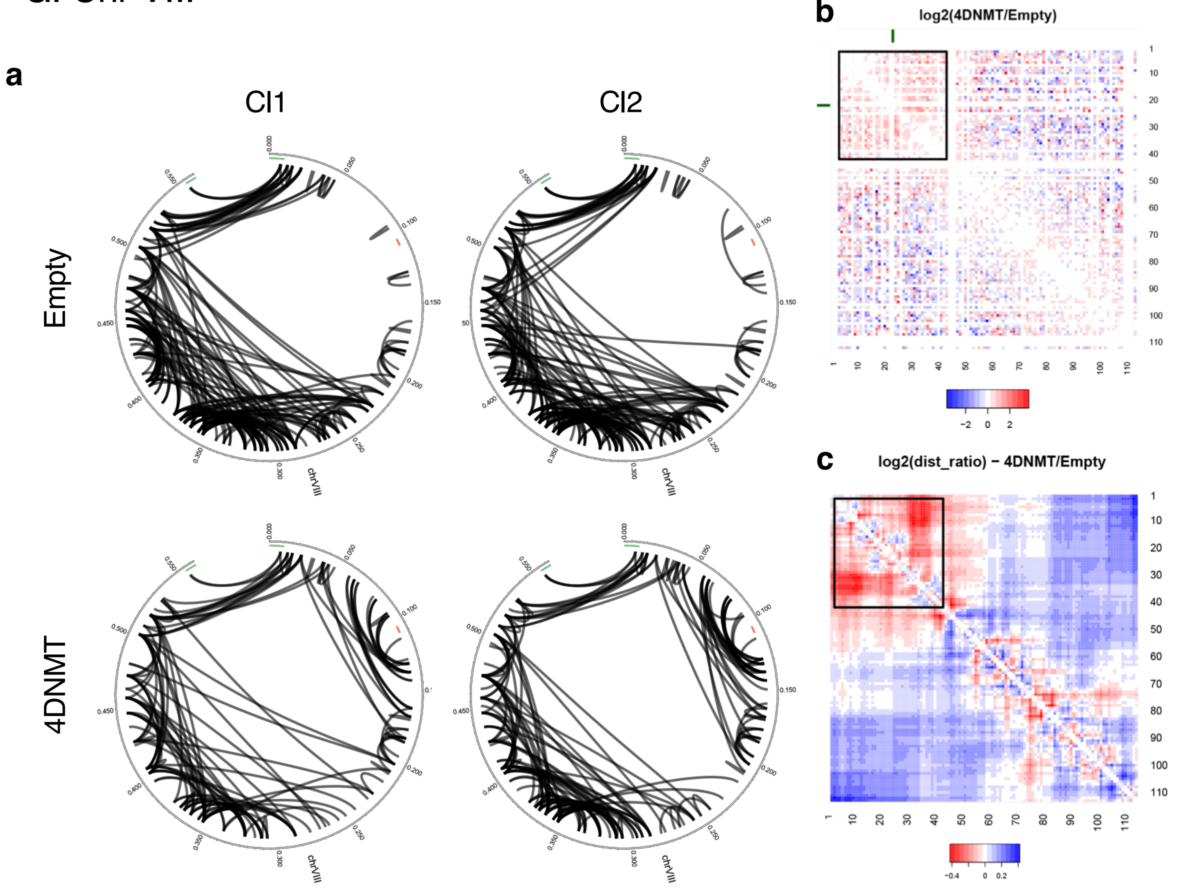

H. Chr IX

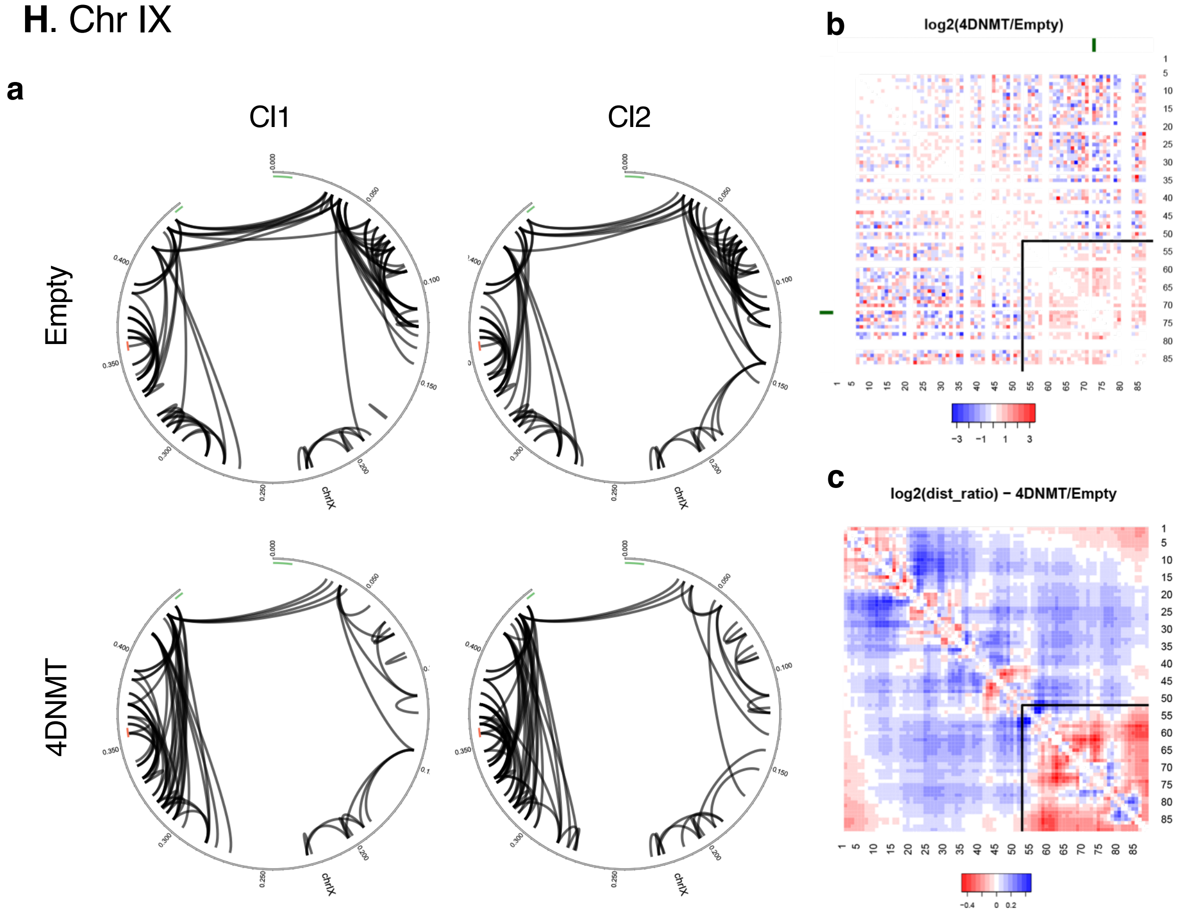

Supplementary Figure 17. Comparison of interactions in the control and the methylated strains

I. Chr X

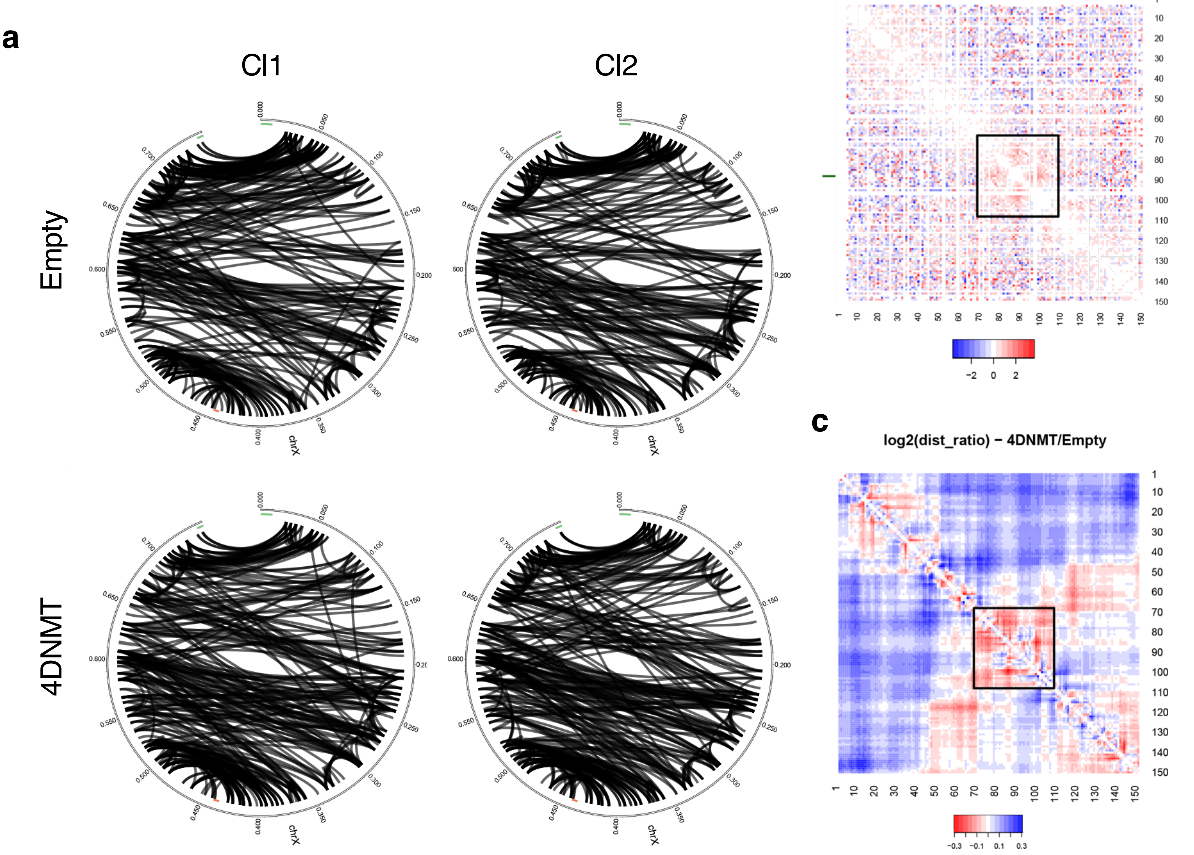

J. Chr XI

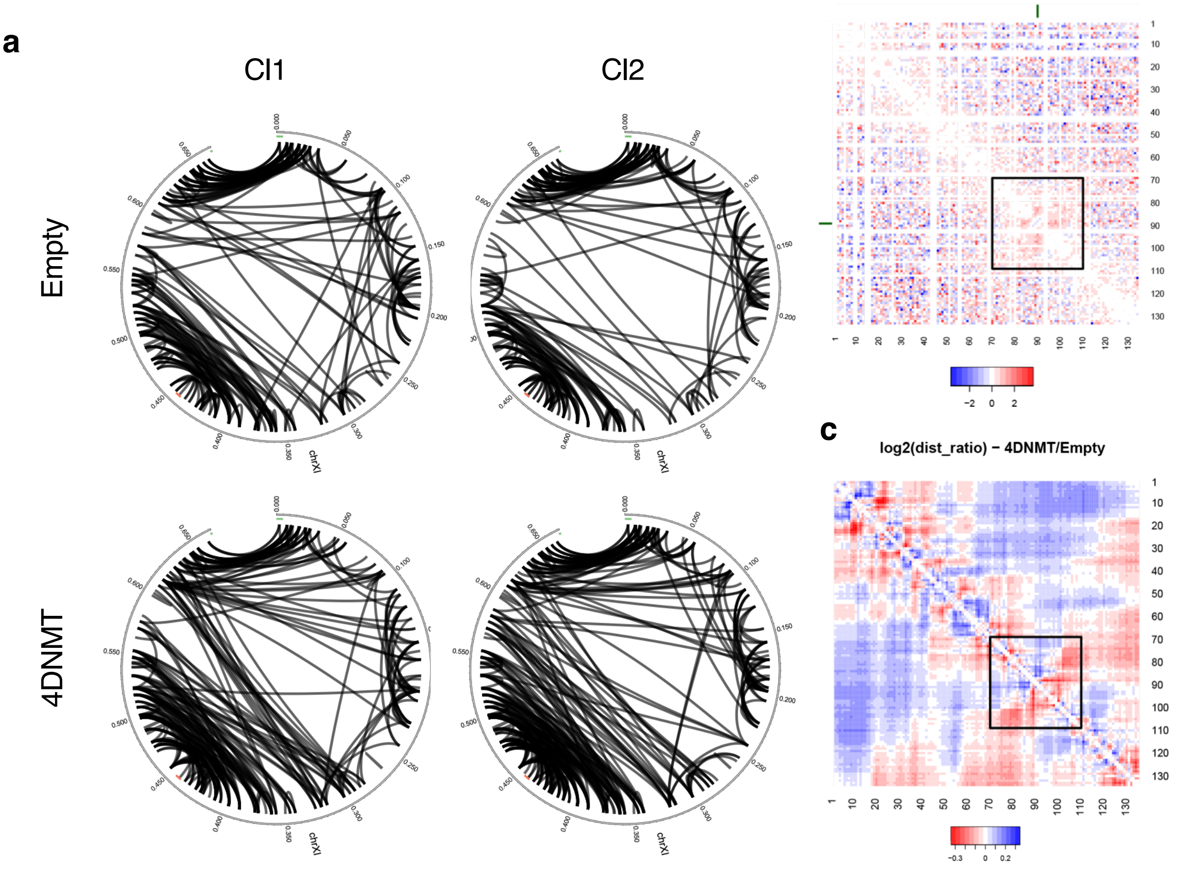

Supplementary Figure 17. Comparison of interactions in the control and the methylated strains

K. Chr XIII

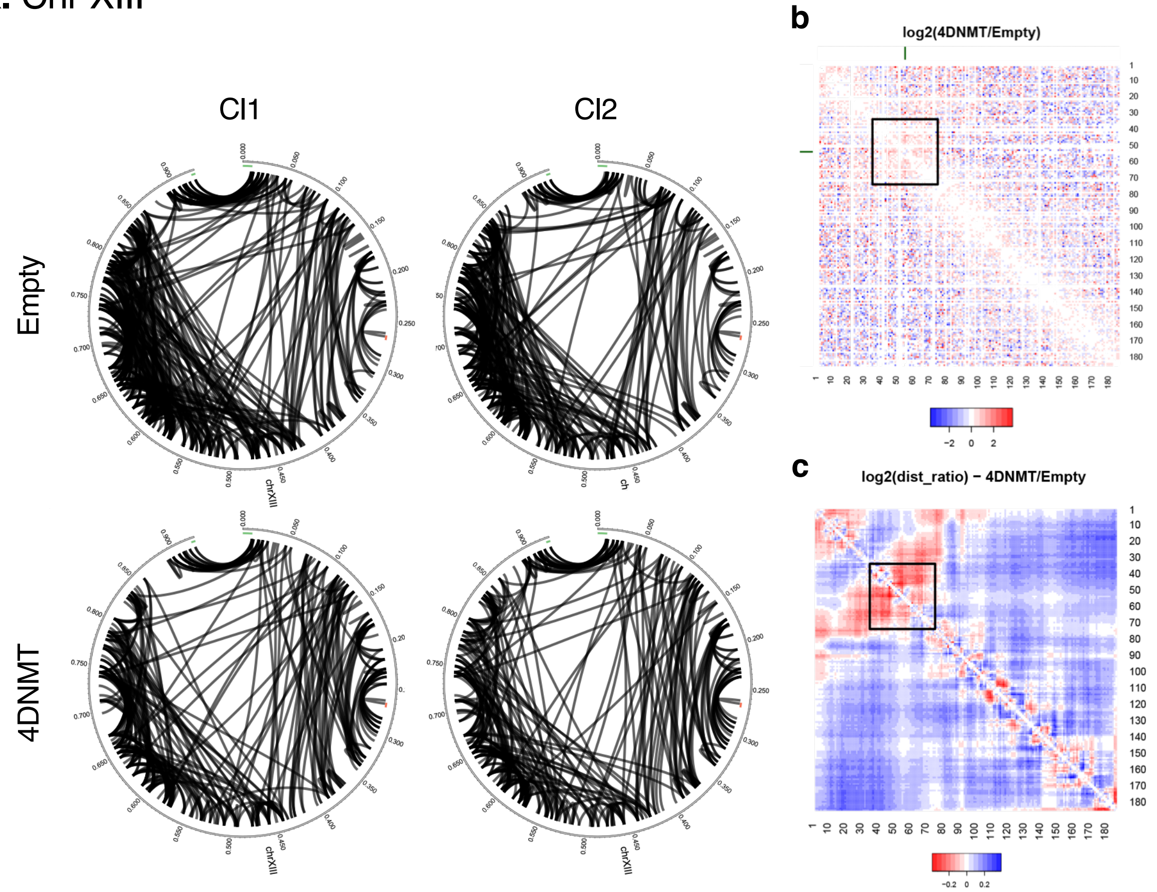

L. Chr XIV

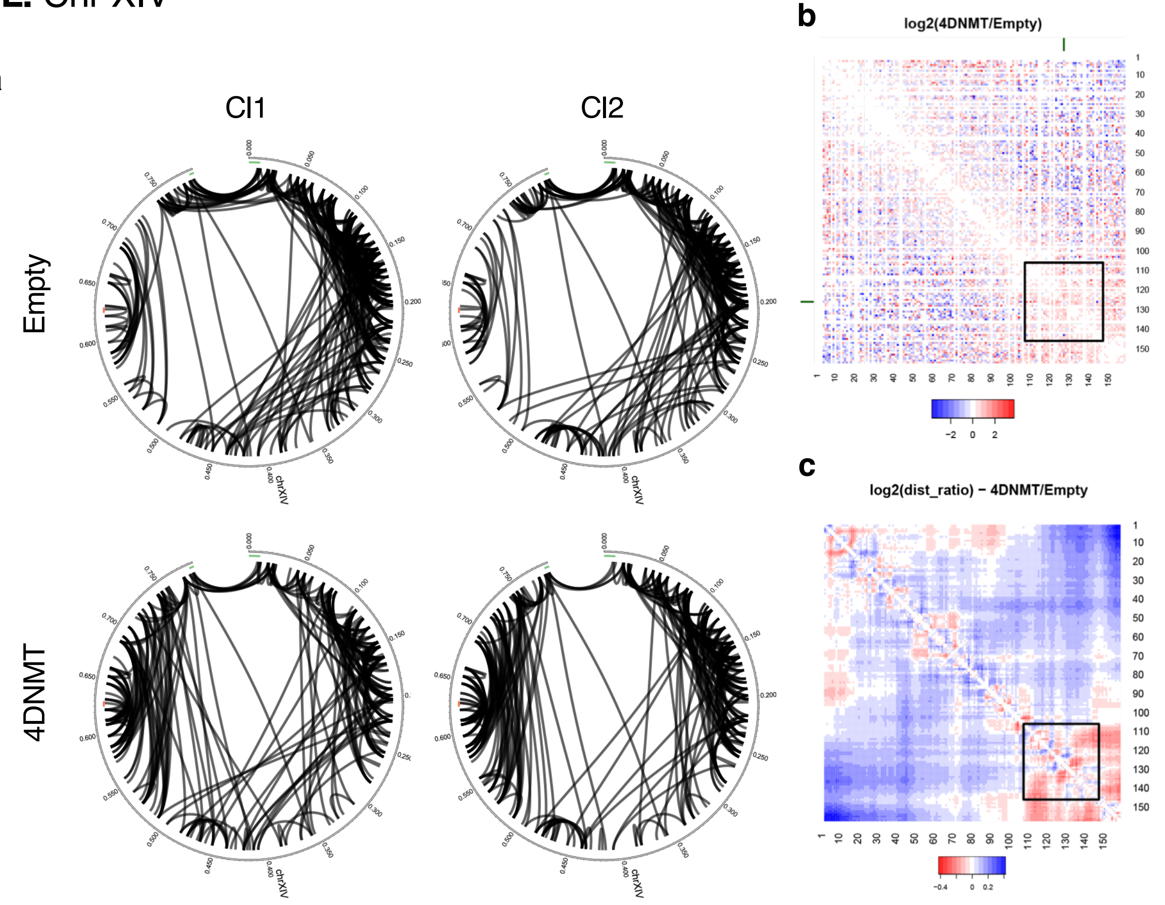

Supplementary Figure 17. Comparison of interactions in the control and the methylated strains

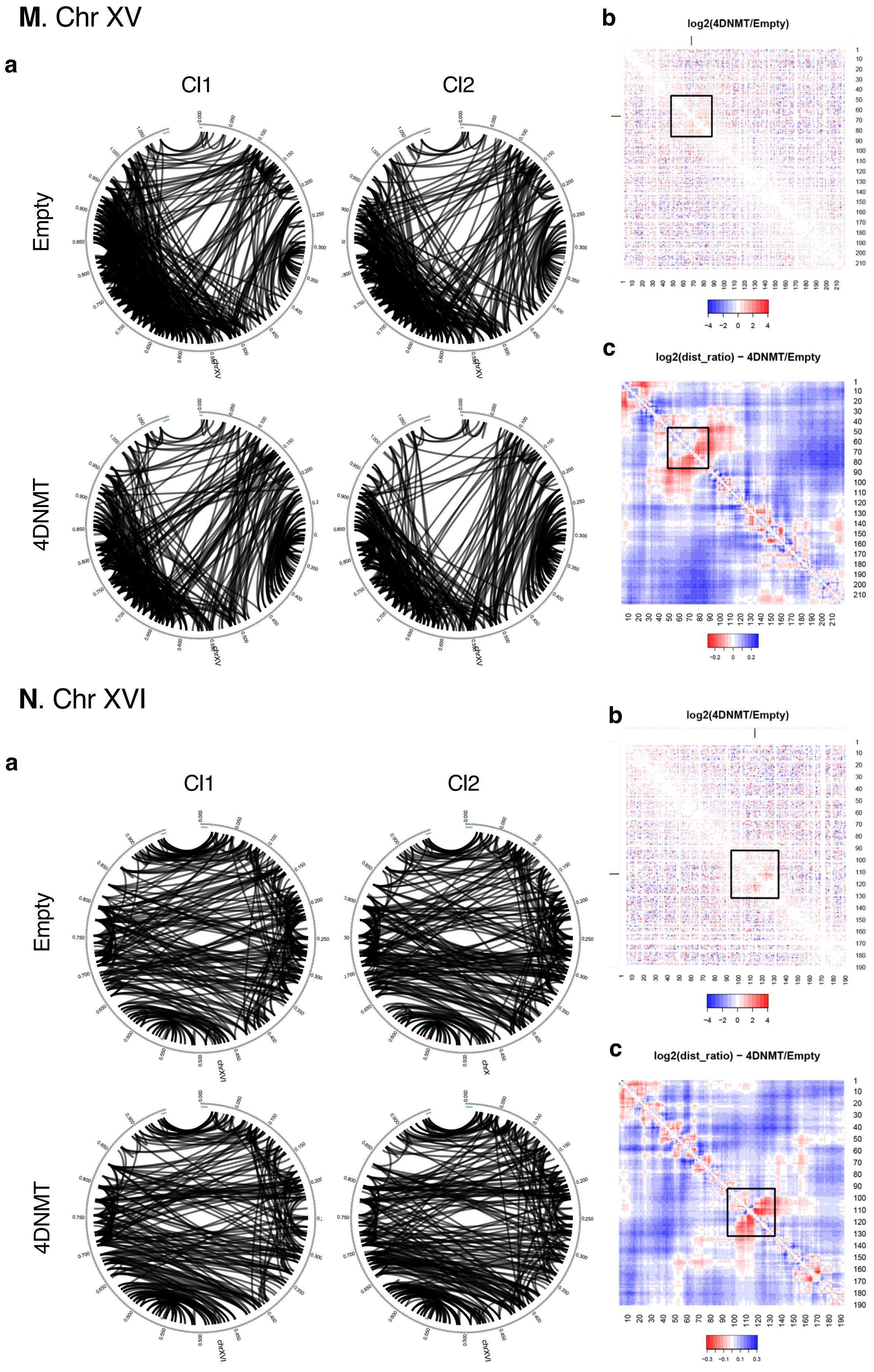

Supplementary Figure 17. Comparison of interactions in the control and the methylated strains

Supplementary Figure 17. Comparison of interactions in the control and the methylated strains. Panels (**A-N**) represent each chromosome (chrI, II, IV-XI and XIII-XIV, respectively). (**a**) Circos diagrams depict each chromosome as a circle. Each arc represents a significant interaction in the control (top) and the methylated sample (bottom) for the two replicas (Cl1, Cl2). The chromosomal position of the centromeres is indicated in red and the telomeres in green. (**b**) Log2 ratio of the interaction frequencies in the control over the methylated for replica 1. Blue indicates interaction with a higher frequency in the control sample and red indicates interactions with a higher frequency in the methylated sample. (**c**) Log2 ratio of the distance in the 3D model for the control over the methylated for replica 1. Blue indicates shorter distance in the control sample and red indicates closer in the methylated sample.

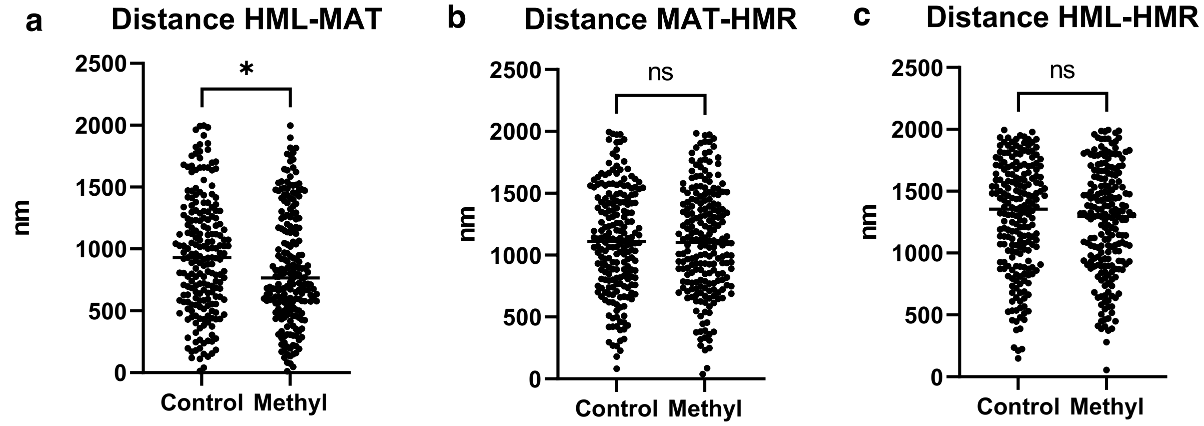

Supplementary Figure 18. Live-Cell Microscopy validates the mating type loci conformation of chromosome III observed in Hi-C. Scatted dot plot showing the intra-nuclear 3D cross-distances between three fluorescent loci in ChrIII in control and methylated cells. Distances between HML and MAT (**a**), MAT and HMR (**b**) and HML and HMR (**c**) were determined by live cell imaging. NS : non significant, * $p \leq 0.05$ (Wilcoxon 2-tailed test). The number of cells analysed were 207 for the empty and 208 for the methylated samples. Source data are provided as a Source Data file

a

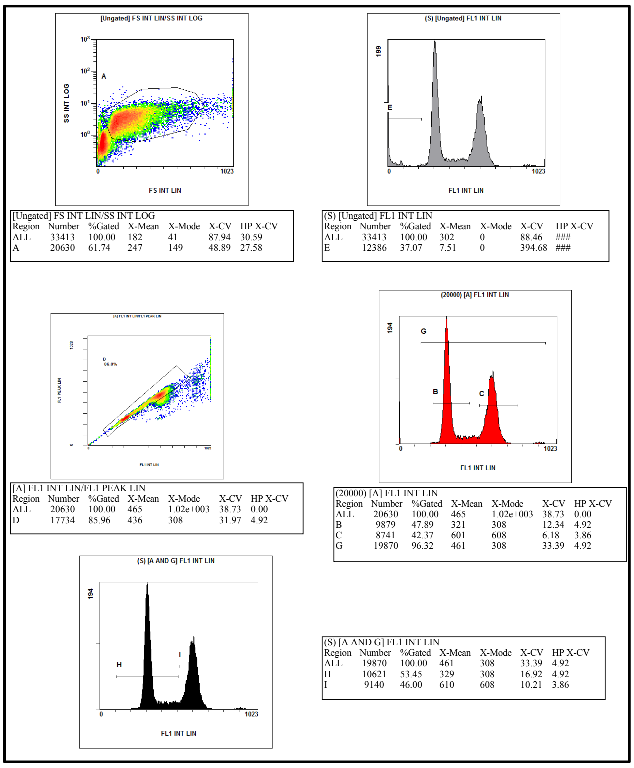
[Ungated] FS INT LIN/SS INT LOG
Region Number %Gated X-Mean X-Mode X-CV HP X-CV
ALL 33413 100.00 182 41 87.94 30.59
A 20630 61.74 247 149 48.89 27.58
(S) [Ungated] FL1 INT LIN
Region Number %Gated X-Mean X-Mode X-CV HP X-CV
ALL 33413 100.00 302 0 88.46 ###
E 12386 37.07 7.51 0 394.68 ###
[A] FL1 INT LIN/FL1 PEAK LIN
Region Number %Gated X-Mean X-Mode X-CV HP X-CV
ALL 20630 100.00 465 1.02e+003 38.73 0.00
D 17734 85.96 436 308 31.97 4.92
(20000) [A] FL1 INT LIN
Region Number %Gated X-Mean X-Mode X-CV HP X-CV
ALL 20630 100.00 465 1.02e+003 38.73 0.00
B 9879 47.89 321 308 12.34 4.92
C 8741 42.37 601 608 6.18 3.86
G 19870 96.32 461 308 33.39 4.92
(S) [A AND G] FL1 INT LIN
Region Number %Gated X-Mean X-Mode X-CV HP X-CV
ALL 19870 100.00 461 308 33.39 4.92
H 10621 53.45 329 308 16.92 4.92
I 9140 46.00 610 608 10.21 3.86

b

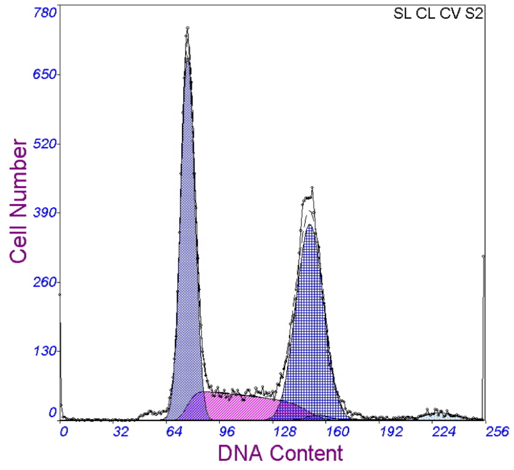
SL CL CV S2
Cell Number
DNA Content

c

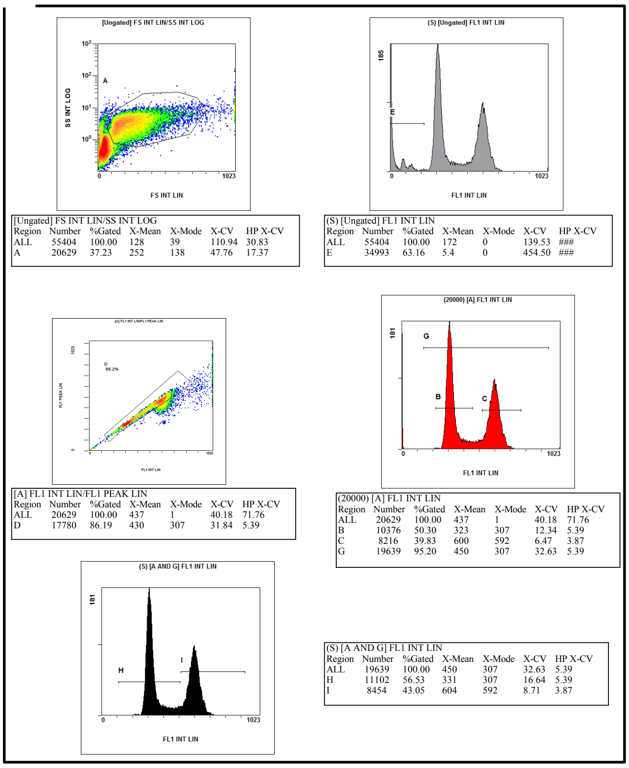
[Ungated] FS INT LIN/SS INT LOG
Region Number %Gated X-Mean X-Mode X-CV HP X-CV
ALL 55404 100.00 128 39 110.94 30.83
A 20629 37.23 252 138 47.76 17.37
(S) [Ungated] FL1 INT LIN
Region Number %Gated X-Mean X-Mode X-CV HP X-CV
ALL 55404 100.00 172 0 139.53 ###
E 34993 63.16 5.4 0 454.50 ###
[A] FL1 INT LIN/FL1 PEAK LIN
Region Number %Gated X-Mean X-Mode X-CV HP X-CV
ALL 20629 100.00 437 1 40.18 71.76
D 17780 86.19 430 307 31.84 5.39
(20000) [A] FL1 INT LIN
Region Number %Gated X-Mean X-Mode X-CV HP X-CV
ALL 20629 100.00 437 1 40.18 71.76
B 10376 50.30 323 307 12.34 5.39
C 8216 39.83 600 592 6.47 3.87
G 19639 95.20 450 307 32.63 5.39
(S) [A AND G] FL1 INT LIN
Region Number %Gated X-Mean X-Mode X-CV HP X-CV
ALL 19639 100.00 450 307 32.63 5.39
H 11102 56.53 331 307 16.64 5.39
I 8454 43.05 604 592 8.71 3.87

d

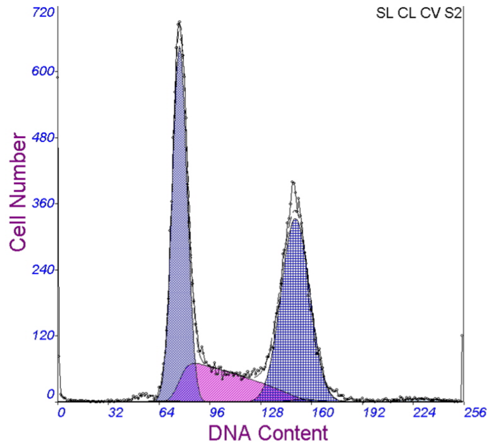
SL CL CV S2
Cell Number
DNA Content

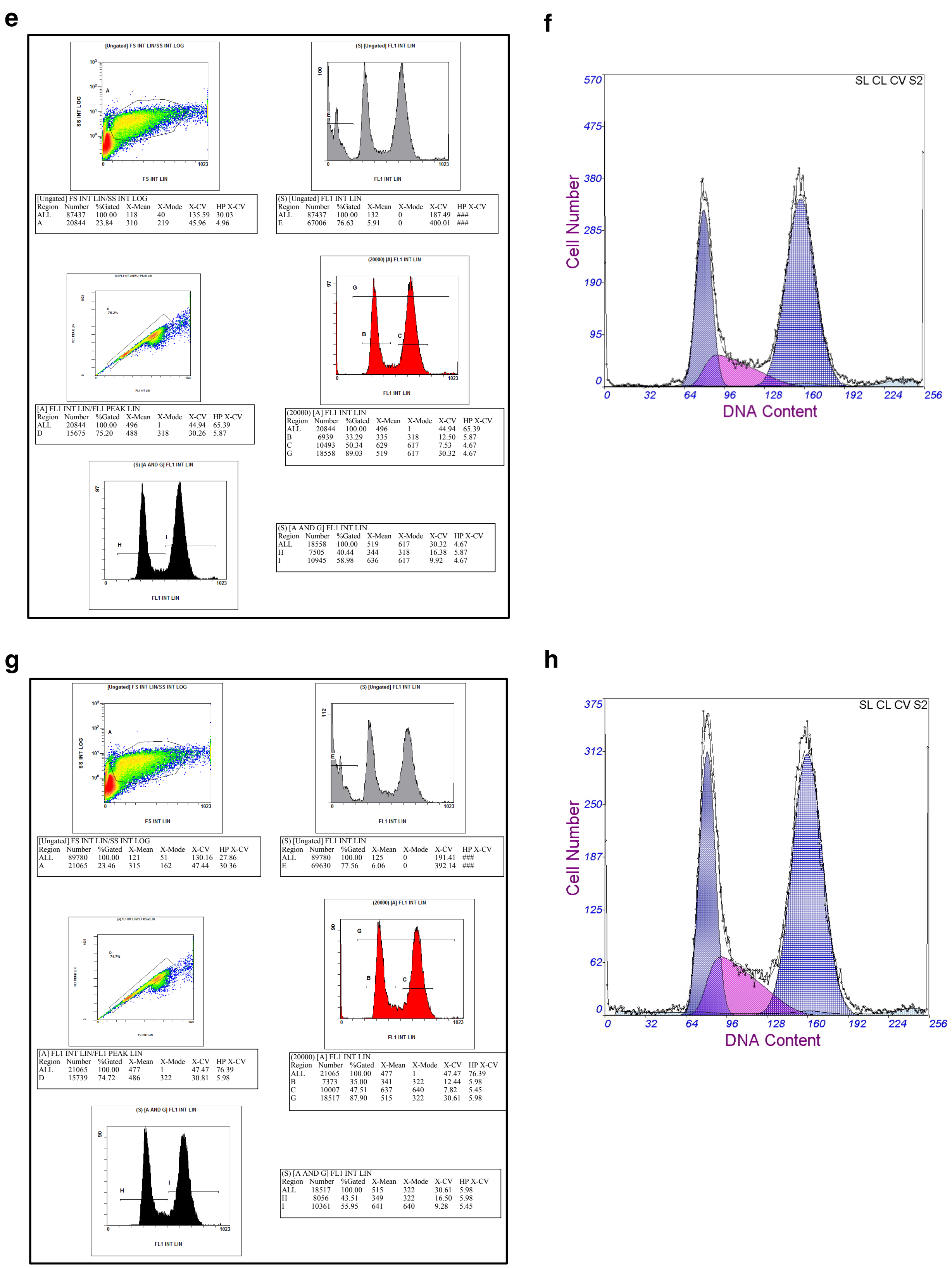

Supplementary Figure 19. Flow Cytometry analysis. (**a,c,e,g**) Gating strategy for cell cycle distribution. (**b,d,f,h**) Graph obtained using MultiCycle software (Phoenix Flow Systems, San Diego, CA, USA) to determine the percentage of cells in each cell cycle phase used to create Supplementary Fig, 2b. (**a,b**) correspond to control 1, (**c,d**) to control 2, **(e,f**) to methylated 1 and (**g,h**) to methylated 2

DNMT Expressed	Hours of induction	State of the culture	Cytosine (ng/ml)	Methyl-Cytosine (ng/ml)	%MeC
Negative control	48 hrs	Exponential	9097.59	0.00	0
DNMT1, 3a, 3b, 3L (Transf 1)	48 hrs	Exponential	4434.69	100.63	2.22
DNMT1, 3a, 3b, 3L (Transf 2)	48 hrs	Exponential	6389.64	133.83	2.05
DNMT1, 3a, 3b, 3L	38 hrs	Saturation	4520.49	193.94	4.11

Supplementary Table 1. Average Cytosine methylation obtained by HPLC/MS

	Exponential Sample			Saturation Sample				Comp 1			Comp 2			Comp 3		
	p(comp1)	p(comp2)	p(comp3)	p(comp1)	p(comp2)	p(comp3)	Log Likelihood Ratio	α	β	E(meth)	α	β	E(meth)	α	β	E(meth)
Model 1: fullmodel	0.000	0.791	0.209	0.000	0.053	0.947	0	2.16	61.40	0.034	2.35	28.10	0.077	3.12	6.67	0.319
Model 2: single component for Saturation sample	0.000	0.769	0.231	0.000	0.000	1.000	-390	2.16	61.40	0.034	2.38	29.20	0.075	2.57	5.87	0.305
Model 3:single component for Exponential sample	0.000	1.000	0.000	0.000	0.056	0.944	-8522	2.16	61.40	0.034	1.20	8.31	0.126	2.93	6.21	0.321
Model 4:single component for both samples	0.000	1.000	0.000	0.000	0.000	1.000	-8709	2.16	61.40	0.034	1.19	8.25	0.126	2.52	5.63	0.309

Supplementary Table 2. Summary of results from the mixture model applied to the nanopore reads.

		Samples in G1			Samples at saturation		
Name gene	Gene ID	Differential expression LOG2FC	p-adj	Methylation level at URS1 site	Differential expression LOG2FC	p-adj	Methylation level at URS1 site
SAE3	YHR079C	6.20	3.38E-21	0.14-0.145	7.43	7.21E-04	0.404-0.37
MEI5	YPL212C	3.84	1.07E-04	0.354-0.318	6.98	1.44E-03	0.509-0.447
GMC2	YLR445W	3.69	5.41E-02	0.176-0.193	6.94	5.98E-03	0.414-0.494
HOP2	YGL033W	2.42	4.26E-03	0.074-0.074	6.83	1.17E-03	0.315-0.392
HED1	YDR014W-A	2.98	3.09E-17	0.203-0.139	4.97	7.21E-04	0.418-0.339
SPO13	YHR014W	4.06	8.93E-07	0.27-0.205	4.95	2.24E-03	0.536-0.554
MEK1	YOR351C	3.23	1.42E-02	0.211-0.181<br>0.354-0.297	4.89	5.47E-03	0.386-0.406<br>0.517-0.476
REC114	YMR133W	3.05	2,32E-2	0.372-0.35	4.84	4.00E-03	0.798-0.811
MER1	YNL210W	2.65	3.06E-01	0.368-0.401	4.75	5.65E-02	0.716-0.749
SPO11	YHL022C	3.12	1.44E-11	0.196-0.222	3.76	3.19E-03	0.727-0.69
DMC1	YER179W	3.08	9.06E-13	0.312-0.212	2.97	1.84E-03	0.469-0443
MEI4	YER044C-A	2.19	4.61E-03	0.165-0.13	2.74	1.52E-02	0.414-0.465
HOP1	YIL072W	1.70	1.34E-03	0.088-0.034	2.55	3.19E-03	0.301-0.262
ZIP1	YDR285W	1.28	1.01E-01	0.125-0.104	2.46	5.21E-03	0.348-0.321
REC102	YLR329W	1.69	2.44E-02	0.311-0.197	2.29	3.66E-08	0.533-0.419
IME2	YJL106W	-0.06	2.60E-01	0.011-0.011	2.27	6.04E-03	0.07-0.08
SPO16	YHR153C	0.65	6.16E-01	0.02-0.043	2.04	1.36E-02	0.224-0.155
REC104	YHR157W	-0.54	4.09E-01	0.039-0.033	1.15	2.50E-02	0.114-0.05
RED1	YLR263W	-0.79	1.87E-02	ND	0.18	5.88E-01	ND
RIM4	YHL024W	2.87	4.84E-17	0.012-0	-0.12	8.22E-01	0.082-0.048

Supplementary Table 3. Expression changes and URS1 methylation level of a subset of early meiotic genes. Differential expression between conditions was performed with DESeq2 with default parameters (Wald two-sided test with Benjamini & Hochberg multiple test correction).

Name	Sequence	Application
F-DNMT3b-mutant	5´ TGGTGGAAGCGTAGACAATGATCTCTCTAACGTCAATC 3´	DNMT3b Mutagenesis
R-DNMT3b-mutant	5´ ATCACCAAGTCGAACGGG 3´	
F-DNMT3A-mutant	5´ TGGAGGCAGTGTCGACAATGACCTCTCCATTG 3´	DNMT3a Mutagenesis
R-DNMT3A-mutant	5´ATCACCAGGTCGAATGGG 3´	
F-DNMT1-mutant	5´ TGGGCCACCCAGCCAGGGCTTCA 3´	DNMT1 Mutagenesis
R-DNMT1-mutant	5´ CCACACAGCATCTCCACATCGCC 3´	
pcrDNMT3b_Kpn1 5´_F	5´GGGGGTACCATGAAGGGAGACAGCAGACATCT 3´	Subcloning DNMT3b cDNA in pBEVY-GU
pcrDNMT3b_EcoRI 3´_R	5´ CTGGATATCTGCAGAATTCCTATTCACAG 3´	
pcrDNMT3L_SacI1_5´_ F	5´ GACGAGCTCCAACAAAATGGACTACAAAGACGATGA 3´	Subcloning DNMT3L cDNA in pBEVY-GT
pcrDNMT3L_EcoRI 3´_R	5´ GTCGAATTCGGTGATTCATTTCTAAAGAGGAAGTG 3´	
UME6-F	5´ GATCCATATGCTAGACAAGGCGCGCTC 3´	Amplification *UME6* coding sequence from yeast genomic DNA
UME6-R	5´ GATCCTCGAGAGTGAGCTTTTATTTTTTTTTCATTGCTC 3´	
F2_pFA6aKan_TRP1	5´ ATTGAGCACGTGAGTATACGTGATTAAGCACACAAAGGCAGCTTGGAGT ATGCGGATCCCCGGGTTAATTAA 3´	Construction yIL30-W strain
R1_pFA6aKan_TRP1	5´ AAAGGCTTGCAGGCAAGTGCACAAACAATACTTAAATAAATACTACTCAG TAATAACCTAGAATTCGAGCTCGTTTAAAC 3´	
Spo13-Cy5-F:	5´ CY5- TTAATTAGGAGTATATTGAGAAATAGCCGCCGACAAAAAGGAAGTCTCA TAAAAGT 3´	UME6 Gel retardation assay
Spo13-R:	5´ ACTTTTATGAGACTTCCTTTTTGTCGGCGGCTATTTCTCAATATACTCCTAA TTAA 3´	
Spo13-Met-Cy5-F:	5´ Cy5- TTAATTAGGAGTATATTGAGAAATAGC(meC)GC(meC)GACAAAAAGGA AGTCTCATAAAAGT 3´	
Spo13-Met-R:	5´ ACTTTTATGAGACTTCCTTTTTGT(meC)GG(meC)GGCTATTTCTCAATATA CTCCTAATTAA 3´	

Supplementary Table 4. Name and sequence of the oligonucleotides used in this study

	Empty-Cl1		Empty-Cl2		4DNTM-Cl1		4DNMT-Cl2	
	Number of reads	(%)	Number of reads	(%)	Number of reads	(%)	Number of reads	(%)
Total reads	77,290,108		84,096,809		63,980,708		60,623,554	
Mapped both	67,901,532	100.00	67,901,533	100.00	55,086,767	100.00	53,623,524	100.00
1- self-circle	1,748,467	2.58	2,190,336	2.96	1,623,463	2.95	1,291,620	2.41
2- dangling-end	9,508,945	14.00	9,601,236	12.98	9,017,932	16.37	6,208,357	11.58
3- error	539,769	0.79	54,4375	0.74	492,032	0.89	552,491	1.03
4- extra dangling-end	17,466,889	25.72	19,224,967	25.99	14,483,116	26.29	15,732,626	29.34
5- too close from RES	8,959,962	13.20	9,812,011	13.26	7,056,667	12.81	7,737,173	14.43
6- too short	1,021,588	1.50	1,120,886	1.52	833,844	1.51	918,430	1.71
7- too large	8,551,118	12.59	9,156,511	12.38	7,686,856	13.95	6,982,601	13.02
8- over-represented	5,154,043	7.59	5,511,705	7.45	4,950,744	8.99	4,553,967	8.49
9- duplicated	31,496,318	46.39	41,497,816	56.09	20,691,442	37.56	20,287,110	37.83
10- random breaks	4,162,523	6.13	4,363,097	5.90	4,007,872	7.28	3,326,301	6.20
Filtered reads	28,515,650	42.00	25,653,808	37.78	25,871,440	46.96	27,031,064	50.41

Supplementary Table 5. Details of the number of excluded reads

Supplementary Method. Mixture model for CpG methylation in nanopore reads.

The CpG methylation calling from the nanopolish software was used to produce for each read the number of CpGs called as being methylated or non-methylated. Let (a_i, b_i) be the methylated and non-methylated counts respectively for read i.

The distribution of (a_i, b_i) can be modelled as a beta-binomial distribution with parameters (α, β). If a read has n CpGs then the expected number of methylated CpGs in the read is given by $n\alpha / (\alpha + \beta)$ and the variance of this number is $n\alpha\beta\ (\alpha + \beta + n) / ((\alpha + \beta)^2(\alpha + \beta + 1))$, so in general higher values of (α, β) give a narrower distribution with a smaller variance.

To model heterogeneity in the methylation distribution across different reads we fit a mixture model with 3 components. Each component has different beta-binomial parameters, so the parameters for component j are. (α_j, β_j) Let p_j be the proportion of reads that belong to component j. The probability of the counts (a_i, b_i) for an individual read is then:

$$\sum_j p_j \frac{(a_i+b_i)!}{a_i!b_i!} \frac{\mathrm{B}(a_i+\alpha_j, b_i+\beta_j)}{\mathrm{B}(\alpha_j, \beta_j)}$$

We estimated the parameters using the EM algorithm for the first component, (α_1, β_1) from the non-methylated samples using a model with only a single component. This allows the modelling of the false positive error from the methylation calling. We then analyzed the methylated samples using a three component model where the first component used the parameters estimated from the non-methylation samples, the second component was a low/medium methylation component and the third component was a high methylation component. The parameters of the 2nd and 3rd components and the mixing proportions $\boldsymbol{p}$ were estimated from the data using the EM algorithm. The parameter estimates for this analysis are given in Table S2 as model 1: full model.

To estimate the statistical support for the multiple components (i.e., the significance of the heterogeneity in read methylation) we tested multiple models by comparing the log likelihood of the different models to the log likelihood under the full model (model 1). For each model the non-fixed parameters were maximized using the EM algorithm. The tested models were:

- Model 2 – The cells in stationary phase were forced to have only a single component (so modelling a homogenous population). The cells in exponential phase were modelled with 3 components as in model 1.
- Model 3 – The cells in exponential phase were forced to have only a single component (so modelling a homogenous population). The cells in stationary phase were modelled with 3 components as in model 1.
- Model 4 – All methylated samples were forced to have a single component, although this component could differ between the exponential and stationary phase samples (i.e., each sample was assumed to be homogenous, but reads from the two sets of samples could come from different populations).

We can consider that when comparing model 1 to models 2 or 3 we have fixed 2 parameters (as we have only 1 component rather than 3 for one of the samples) so twice the log likelihood difference between the models is distributed as a X^2 with 2 degrees of freedom. Similarly, twice the log likelihood difference between models 2 and 3 and model 4 is also distributed as a X^2 with 2 degrees of freedom.
