# Supplementary material for: Impact of DNA methylation on 3D genome structure
Source: Nat Commun. 2021 May 28;12:3243. doi: 10.1038/s41467-021-23142-8 (PMC8163762; doi:10.1038/s41467-021-23142-8)
Supplement: Supplementary file 2 — Reporting Summary [file 41467_2021_23142_MOESM2_ESM.pdf]

Corresponding author(s): Modesto Orozco and Isabelle Brun Heath

Last updated by author(s): Mar 9, 2021

Reporting Summary

Nature Research wishes to improve the reproducibility of the work that we publish. This form provides structure for consistency and transparency in reporting. For further information on Nature Research policies, see our Editorial Policies and the Editorial Policy Checklist.

Statistics

For all statistical analyses, confirm that the following items are present in the figure legend, table legend, main text, or Methods section.

n/a	Confirmed	
☐	☒	The exact sample size (*n*) for each experimental group/condition, given as a discrete number and unit of measurement
☐	☒	A statement on whether measurements were taken from distinct samples or whether the same sample was measured repeatedly
☐	☒	The statistical test(s) used AND whether they are one- or two-sided<br>*Only common tests should be described solely by name; describe more complex techniques in the Methods section.*
☒	☐	A description of all covariates tested
☐	☒	A description of any assumptions or corrections, such as tests of normality and adjustment for multiple comparisons
☐	☒	A full description of the statistical parameters including central tendency (e.g. means) or other basic estimates (e.g. regression coefficient) AND variation (e.g. standard deviation) or associated estimates of uncertainty (e.g. confidence intervals)
☐	☒	For null hypothesis testing, the test statistic (e.g. *F*, *t*, *r*) with confidence intervals, effect sizes, degrees of freedom and *P* value noted<br>*Give P values as exact values whenever suitable.*
☒	☐	For Bayesian analysis, information on the choice of priors and Markov chain Monte Carlo settings
☒	☐	For hierarchical and complex designs, identification of the appropriate level for tests and full reporting of outcomes
☐	☒	Estimates of effect sizes (e.g. Cohen's *d*, Pearson's *r*), indicating how they were calculated

Our web collection on statistics for biologists contains articles on many of the points above.

Software and code

Policy information about availability of computer code

Data collection

Data collection did not require the use of any software.

Data analysis

Programs for reconstruction and visualization of chromatin structure (from nucleosome to entire chromatin) are available at the H2020 MuG virtual research environment (https://www.multiscalegenomics.eu/)
The links, when available, to all the softwares used in this study are listed hereafter.
Nucleosome Dynamics/nucleR
Used to determine nucleosome positioning and nucleosome dynamics
https://github.com/nucleosome-dynamics

TADbit
Used to analyse Hi-C data and set up interaction maps
https://github.com/3DGenomes/TADbit

Juicebox
Juicebox version 1.11.08 was used to display interaction maps from the data obtained with TADbit.
https://github.com/aidenlab/Juicebox/wiki/Download

diffHic
Used to detect changes between 2 Hi-C interaction matrices.
https://www.bioconductor.org/packages/release/bioc/html/diffHic.html

Hi-C derived chromatin model
Software required:

Amber Molecular Dynamics Package (licensed).
Version: Amber 18
Implementation: PMEMD implementation of SANDER for GPU (pmemd.cuda)
Web: http://ambermd.org

AmberTools (free).
Version: AmberTools 18
Web: http://ambermd.org

VMD
Version: VMD for LINUXAMD64, version 1.9.3 (November 30, 2016)
Web: http://www.ks.uiuc.edu/Research/vmd

Usage:
From a PDB file consisting in an appropriate number of beads (according to Hi-C map binning) located in random positions, the input topology and starting structure was obtained with the tLEap module from AmberTools. Distance restraints were applied as the NMR restraints implemented in Amber. The restraint file (RST) was built from the distance matrix previously derived from HiC contact map (see Methods). Equilibrium and production simulations were performed applying the distance restraints, using nmropt=1 option and the RST file previously mentioned. The output is a netcdf file containing the trajectory of the simulated structures. It was visualized with VMD software and further analyzed with cpptraj from AmberTools.

Chromatin coarse-grained model at the nucleosome level
- A compressed file (Chromatin_Dynamics.zip) with the software is available as Supplementary Software. Follow instructions from the README file.
- More information can be found in the corresponding "Code and Software Submission Checklist" (nr-software-policy-chromatin-dynamics.pdf).

WEB VERSION
Chromatin dynamics can be executed on a web server:
http://vre.multiscalegenomics.eu/launch/

Whole Genome Bisulfite sequencing analysis
Alignment and Methylation calling were performed using gemBS version 3.0
https://github.com/heathsc/gemBS

Nanopore analysis
Quality control was monitored using MinKNOW 1.15.4
Base calling was performed using Guppy 2.3.7.
Reads were mapped using minimap2 2.9-r720 and CpG methylation was called using nanopolish 0.11.0.
The code used to identify the differentially methylated population of nanopore reads is provided as a tar file (nanopore_paper.tar) with the program, 3 test data files and a short README, available as Supplementary Software.
More information can be found in the corresponding "Code and Software Submission Checklist" (nr-software-policy-nanopore.pdf).

Image Analysis
ImageJ Fiji (Version 1.53) is available at https://imagej.net/Fiji/Downloads
Spot distance plugin (Version 08.2014, Daniel Sage EPFL) on ImageJ Fiji distribution is available at
http://bigwww.epfl.ch/sage/soft/spotdistance/

ChIP-seq analysis
ChIP-seq data were analysed with BWA and MACS2 integrated with the European UseGalaxy server (https://usegalaxy.eu/)

For manuscripts utilizing custom algorithms or software that are central to the research but not yet described in published literature, software must be made available to editors and reviewers. We strongly encourage code deposition in a community repository (e.g. GitHub). See the Nature Research guidelines for submitting code & software for further information.

Data

Policy information about availability of data

All manuscripts must include a data availability statement. This statement should provide the following information, where applicable:

- Accession codes, unique identifiers, or web links for publicly available datasets
- A list of figures that have associated raw data
- A description of any restrictions on data availability

WGBS, RNA-seq, Mnase-seq and Hi-C raw data have been submitted to the European Nucleotide Archive (ENA) under accession number E-MTAB-9258, E-MTAB-9195, E- MTAB-9259 and E-MTAB-9257 respectively. Nanopore processed data as well as Fastq data have been submitted to the European Nucleotide Archive (ENA) under accession number E-MTAB-9356. ChIP-seq raw and processed data have been submitted to the European Nucleotide Archive (ENA) under accession number E-MTA-10001.

Field-specific reporting

Please select the one below that is the best fit for your research. If you are not sure, read the appropriate sections before making your selection.

☒ Life sciences ☐ Behavioural & social sciences ☐ Ecological, evolutionary & environmental sciences

For a reference copy of the document with all sections, see nature.com/documents/nr-reporting-summary-flat.pdf

Life sciences study design

All studies must disclose on these points even when the disclosure is negative.

Sample size	2 conditions tested (control or methylated) at two different growth stage (Exponential or Stationary)
Data exclusions	No data were excluded
Replication	2 biological replicates of each conditions were performed
Randomization	The study was designed to compare the yeast genome conformation between a control sample and a methylated sample so the randomization does not apply in this case.
Blinding	The study was designed to compare the genome conformation between a control sample and a methylated sample so we needed to know what were the results for each condition. The blinding does not apply in this case.

Reporting for specific materials, systems and methods

We require information from authors about some types of materials, experimental systems and methods used in many studies. Here, indicate whether each material, system or method listed is relevant to your study. If you are not sure if a list item applies to your research, read the appropriate section before selecting a response.

Materials & experimental systems

n/a	Involved in the study	
☐	☒	Antibodies
☐	☒	Eukaryotic cell lines
☒	☐	Palaeontology and archaeology
☒	☐	Animals and other organisms
☒	☐	Human research participants
☒	☐	Clinical data
☒	☐	Dual use research of concern

Methods

n/a	Involved in the study	
☐	☒	ChIP-seq
☐	☒	Flow cytometry
☒	☐	MRI-based neuroimaging

Antibodies

Antibodies used	anti-DNMT1 (ref ab87654, Abcam)<br>anti-DNMT3a (ab2850, Abcam)<br>anti-DNMT3b (ab122932, Abcam)<br>anti-Flag (F7425, Sigma)<br>anti Rabbit (Goat)-HRP conjugated (65-6120, Invitrogen)<br>H3K4me1 (ab8895, Abcam)<br>H3K4me3 (ab8580, Abcam)
Validation	Antibodies were validated by the manufacturers. Species and application specificities are available on their respective website.

Eukaryotic cell lines

Policy information about cell lines

Cell line source(s)	The YHP499 Yeast strain used in this study was purchased from ATCC (Saccharomyces cerevisiae Meyen ex E.C. Hansen (ATCC® 76625™)<br>The yIL30 strain (Mata ade2-1 can1-100 his3-11, 15 leu2-3, 112 trp1-1 ura3-1 ade2-1::His3p-CFP-lacI-URA3p-λcIYFP-ADE2, TetR-mRFP:NAT1, MAT5':: λO-HIS, HMR:: LacO-TRP, HML::TetO-LEU) carrying the three integration sites for the fluorescent tags was kindly provided by Kerstin Bystricky.
Authentication	The auxotrophies of the strains were tested to confirm the genotype

Mycoplasma contamination | Mycoplasma contamination is not relevant for yeast cultures

Commonly misidentified lines (See ICLAC register) | *Name any commonly misidentified cell lines used in the study and provide a rationale for their use.*

ChIP-seq

Data deposition

☒ Confirm that both raw and final processed data have been deposited in a public database such as GEO.

☒ Confirm that you have deposited or provided access to graph files (e.g. BED files) for the called peaks.

Data access links<br>*May remain private before publication.*	E-MTAB-10001
Files in database submission	H3K4me1 and H3K4me3 ChIPseq in control and unmethylated strains
Genome browser session<br>(e.g. UCSC)	Not applicable

Methodology

Replicates	2 biological replicates of each conditions were performed
Sequencing depth	The libraries from ChIPseq were done using the NEBNext Ultra DNA Library Prep kit for Illumina (Ref.:#7370) following the manufacturer protocol and sequenced at 1x50bp.
Antibodies	H3K4me1 (ab8895, Abcam), H3K4me3 (ab8580, Abcam)
Peak calling parameters	Peak calling was performed using MACS2 with default parameters.
Data quality	Fragments with MAPQ quality score below 20 were discarded.
Software	Sequenced reads were analyzed using the MACS2 available in the Galaxy platform

Flow Cytometry

Plots

Confirm that:

☐ The axis labels state the marker and fluorochrome used (e.g. CD4-FITC).

☐ The axis scales are clearly visible. Include numbers along axes only for bottom left plot of group (a 'group' is an analysis of identical markers).

☐ All plots are contour plots with outliers or pseudocolor plots.

☒ A numerical value for number of cells or percentage (with statistics) is provided.

Methodology

Sample preparation	*Describe the sample preparation, detailing the biological source of the cells and any tissue processing steps used.*
Instrument	Beckam Coulter GalliosTM flow cytometer.
Software	FlowJo and M-Cycle
Cell population abundance	*Describe the abundance of the relevant cell populations within post-sort fractions, providing details on the purity of the samples and how it was determined.*
Gating strategy	*Describe the gating strategy used for all relevant experiments, specifying the preliminary FSC/SSC gates of the starting cell population, indicating where boundaries between "positive" and "negative" staining cell populations are defined.*

☒ Tick this box to confirm that a figure exemplifying the gating strategy is provided in the Supplementary Information.
