# Supplementary material for: Impact of DNA methylation on 3D genome structure
Source: Nat Commun. 2021 May 28;12:3243. doi: 10.1038/s41467-021-23142-8 (PMC8163762; doi:10.1038/s41467-021-23142-8)
Supplement: Supplementary file 4 — Description of Additional Supplementary Files [file 41467_2021_23142_MOESM4_ESM.pdf]

Description of Additional Supplementary Files

File Name: Supplementary Data 1
Description: Table with detailed differential expression results

File Name: Supplementary Software 1
Description: Software for fitting mixture model for methylation frequency to nanopore reads

File Name: Supplementary Software 2
Description: Chromatin Dynamics analysis software
