# Supplementary material for: Impact of DNA methylation on 3D genome structure
Source: Nat Commun. 2021 May 28;12:3243. doi: 10.1038/s41467-021-23142-8 (PMC8163762; doi:10.1038/s41467-021-23142-8)
Supplement: Supplementary file 8 — Source Data [file 41467_2021_23142_MOESM8_ESM.zip › Source Data/SuppFig1_Uncropped.pptx]

## Slide 1
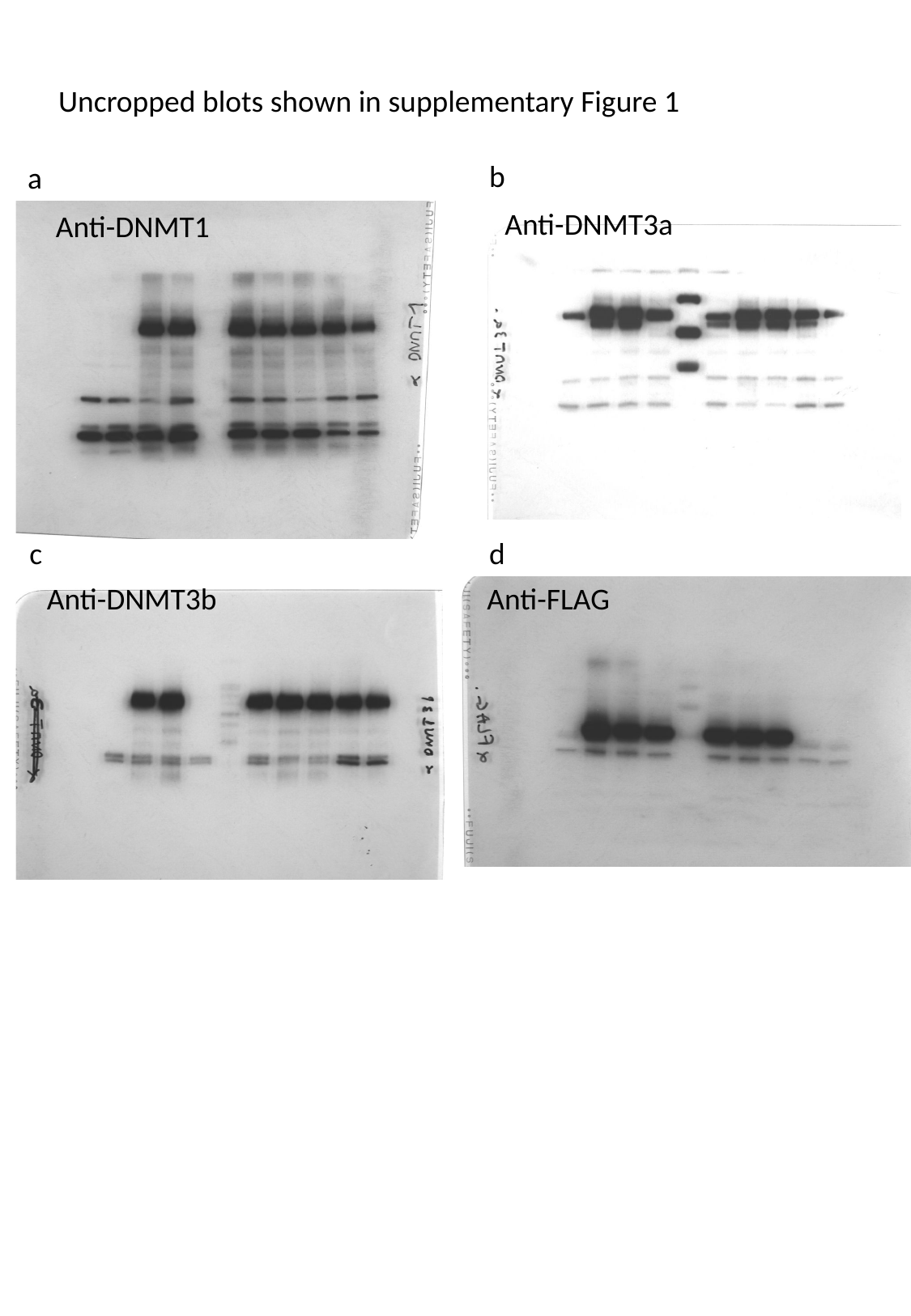

Uncropped blots shown in supplementary Figure 1
b
a
Anti-DNMT3a
Anti-DNMT1
c
d
Anti-DNMT3b
Anti-FLAG
